# 3D Bioprinted Fat‐Myocardium Model Unravels the Role of Adipocyte Hypertrophy in Atrial Dysfunction

**DOI:** 10.1002/advs.202516114

**Published:** 2026-02-08

**Authors:** Lara Ece Celebi, Pinar Zorlutuna

**Affiliations:** ^1^ Department of Aerospace and Mechanical Engineering University of Notre Dame Notre Dame Indiana USA; ^2^ Bioengineering Graduate Program University of Notre Dame Notre Dame Indiana USA; ^3^ Department of Chemical and Biomolecular Engineering University of Notre Dame Notre Dame Indiana USA; ^4^ Harper Cancer Research Institute University of Notre Dame Notre Dame Indiana USA

**Keywords:** 3D bioprinting, adipocyte hypertrophy, cardiac tissue engineering, engineered disease models, obesity‐induced cardiac dysfunction

## Abstract

Cardiovascular diseases (CVD) are the leading cause of mortality in individuals with obesity. Epicardial adipose tissue (EAT) dysfunction serves as a link between obesity and CVD, promoting inflammatory and metabolic alterations that increase CVD risk. While EAT normally supports cardiac health, obesity‐induced adipocyte hypertrophy triggers excessive fatty acid and cytokine release, driving myocardial lipotoxicity and inflammation that impair electrophysiology and metabolism, leading to beating irregularities, insulin resistance, and heart failure. The lack of sufficient EAT in small animal models and the impracticality of using large mammals hinder insights into the effects of EAT hypertrophy on the myocardium. To address this gap, a human‐derived 3D bioprinted coculture of obese adipocytes and cardiomyocytes (CMs) is developed using patient‐derived adipocytes and human induced pluripotent stem cell (hiPSC)‐derived atrial CMs (a‐iCMs). This platform enables the investigation of both cell‐cell and paracrine interactions between hypertrophic adipocytes and a‐iCMs, allowing assessment of electrophysiological, structural, and proteomic changes to uncover mechanisms linking EAT hypertrophy to obesity‐related atrial dysfunction. Screening of metformin, a cardioprotective drug, reveals improvement in electrophysiological function in hypertrophic adipocyte–a‐iCM cocultures. 3D bioprinted fat–myocardium model provides a high‐throughput platform to study obesity‐induced atrial dysfunction and facilitate the discovery of therapies for the obese heart.

## Graphical abstract

A human‐derived 3D bioprinted fat–myocardium model is developed to investigate how adipocyte hypertrophy drives atrial dysfunction in obesity. Palmitate‐induced adipocyte hypertrophy promotes adipose dysfunction that impairs atrial cardiomyocyte metabolism and electrophysiology through both paracrine and direct interactions. This platform recapitulates key features of obese atrial pathology and enables therapeutic screening, as demonstrated by metformin‐mediated functional rescue.

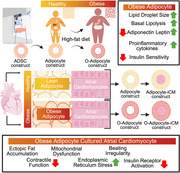

## 1 Introduction

Obesity has reached pandemic levels by affecting over 1 billion people, equivalent to one in eight worldwide [1]. Cardiovascular diseases (CVD) are the leading comorbidities associated with a high body mass index (BMI) and are responsible for two‐thirds of obesity‐related deaths [2]. Cardiac adipose tissue dysfunction serves as a link between obesity and CVD, mediating the inflammatory and metabolic alterations that increase CVD risk in obese patients [3]. Unlike other visceral fat depots, epicardial adipose tissue (EAT) lacks distinct boundaries with neighboring tissue [4] and shares a direct microcirculation with the myocardium. The strategic location of EAT supports cardiac function by facilitating dynamic interactions with the myocardium, including the efficient supply of free fatty acids (FFA), responsible for over 60% of the energy required for healthy heart contraction [5]. It also secretes adipokines that regulate endothelial function [6], exert anti‐inflammatory [7], insulin‐sensitizing [6] and anti‐atherosclerotic effects [8], and contribute to cardioprotection through paracrine and vasocrine signaling mechanisms. However, in obesity, EAT's strategic location becomes a critical liability, as its expansion (via hyperplasia and hypertrophy) and dysfunction lead to the excessive release of FFAs and proinflammatory mediators directly into the myocardium, disrupting metabolic homeostasis and contributing to lipotoxicity, inflammation, and cardiac dysfunction [9, 10]. While healthy adipose tissue expansion favors hyperplasia, which is typically accompanied by a proportional angiogenic response, appropriate extracellular matrix (ECM) remodeling, and minimal inflammation [17], hypertrophic expansion drives obesity‐associated metabolic dysfunction [11]. Hypertrophic adipocytes are characterized by several hallmarks: enlarged cell size due to excessive lipid accumulation [12], increased secretion of proinflammatory cytokines [13], a shift in adipokine profile [14], impaired insulin signaling [15], and altered lipolysis [16], as well as mitochondrial dysfunction [17]. These changes promote ectopic fat accumulation in the myocardium [18], metabolic dysfunction and inflammatory signaling within myocardial cells [19], leading to insulin resistance [20], arrhythmias [21, 22, 23, 24], myocardial fibrosis [25, 26, 27], and atherosclerosis [28], culminating in heart failure [29].

Most clinical studies linking EAT hypertrophy to CVD have utilized imaging techniques such as computed tomography and magnetic resonance imaging [30]. While useful for associating risk factors, these imaging studies often lack mechanistic insights into disease progression since they are primarily retrospective [31]. To bridge this gap, several mechanistic explanations have been proposed utilizing in vitro and in vivo platforms. Venteclef et al. investigated the paracrine effects of human EAT on rat cardiomyocytes (CMs) utilizing an organo‐culture model of rat atria and demonstrated that adipo‐fibrokines released by EAT can promote myocardial fibrosis [25]. This finding suggests that EAT may actively contribute to adverse cardiac remodeling by modulating the local myocardial environment through paracrine signaling. However, small lab animals have limitations in modeling the contractile function of the human heart due to their small size and short lifespan [32]. More importantly, there are notable differences in cardiac fat depots, as most commonly used small lab animals, mice and rats, possess minimal EAT, which is primarily restricted to the atrioventricular groove, and with no direct contact with the myocardium [33], which limits their translational relevance. In contrast, large animal models such as pigs and sheep more closely mimic human EAT distribution and cardiac physiology. Despite their physiological relevance, large animal models are low‐throughput and costly, limiting their utility for mechanistic studies and early‐stage therapeutic screening [34]. Therefore, scalable human‐derived in vitro systems are critical to complement and inform animal studies in the search for effective therapeutics [35].

Human induced pluripotent stem cells (hiPSCs) provide physiologically relevant platforms that potentially enable mechanical investigations, personalized approaches for drug testing, and cytotoxicity assessments. Recently, researchers cocultured adipose‐derived stromal cells (ADSC)‐derived human adipocytes with hiPSC‐derived ventricular CMs (v‐iCMs) in 2D settings [36]. They observed prolonged action potential duration, extended calcium transients, reduced conduction velocity, and increased conduction velocity heterogeneity, along with altered levels of ion channel and gap junction genes in 2D v‐iCMs co‐cultured with adipocytes or exposed to adipocyte‐conditioned medium. This study also demonstrated that paracrine signals from adipocytes alone are sufficient to induce electrophysiological dysfunction. However, 2D cultures of adipocytes and CMs have some inherent limitations. In 2D, adipocytes often lose their natural spherical shape, which can affect gene expression, lipid storage, and adipokine secretion [37]. CMs in monolayer lack the maturation seen in native cardiac tissue, limiting their ability to accurately replicate physiological contraction and electrophysiology [38]. Additionally, without the ECM architecture found in vivo, 2D systems may not fully capture the complex cell‐cell and cell‐matrix interactions involved in adipose tissue dysfunction in obesity. Moreover, the adipocytes used in existing monolayer coculture models with CMs [36, 39, 40] have yet to recapitulate the dysfunctional phenotype characteristic of obesity.

Chamber specificity is crucial for modeling cardiac pathologies, as each heart chamber has distinct structural, functional, and electrophysiological characteristics that influence the manifestation and progression of CVD [41]. However, most hiPSC‐derived engineered models have predominantly focused on generating ventricular myocardium, overlooking the need for atrial myocardium models [42]. Obesity is associated with a 50% increase in the risk of developing atrial fibrillation [43]. Therefore, there is an urgent need for engineered tissues that accurately replicate atrial dysfunction in obesity, providing chamber‐specific mechanistic insights and facilitating the development of targeted therapeutic strategies. Here, we present a human‐derived 3D adipocyte‐hiPSC‐derived atrial CM (a‐iCM) model designed to enable precise quantification of both contact‐dependent (juxtacrine) and paracrine interactions within the context of obesity, which is crucial for the development of targeted and effective therapeutic interventions aimed at treating obesity‐related heart failure. As reported recently [35], there is no existing literature on 3D coculture systems combining adipocytes and CMs. Therefore, this represents the first 3D coculture system combining adipocytes and CMs, established using human patient‐derived cells and palmitate (PA)‐induced hypertrophy to recapitulate the obese adipocyte phenotype to uncover mechanisms linking EAT dysfunction to atrial impairment in obesity.

We previously employed extrusion‐based 3D bioprinting and gelatin methacryloyl (GelMA)‐Collagen type I bioink to model (patho)physiological characteristics of ventricular myocardium [44]. Here, we once again utilized 3D bioprinting and the characterized GelMA‐Collagen type 1 bioink [44] to engineer 3D cultures of healthy (Adi) and PA‐induced hypertrophic (obese‐like, OAdi) ADSC‐derived adipocytes. We characterized the major hallmarks of hypertrophic adipocytes and their paracrine effects on mitochondrial and glycolytic function as well as ATP production in a‐iCMs. Next, we 3D bioprinted a‐iCMs within Adi and OAdi constructs, creating a 3D coculture. Using this coculture model, we reported the effects of 3D Adi/OAdi on a‐iCMs, examining changes in electrophysiology, morphology, and protein expressions related to atrial dysfunction in obesity.

Metformin is an antidiabetic drug that lowers blood glucose levels by activating AMP‐activated protein kinase (AMPK), which suppresses hepatic gluconeogenesis, enhances insulin sensitivity, and promotes glucose uptake in peripheral tissues [45]. To demonstrate the utility of the model for therapeutic screening, we evaluated metformin's ability to mitigate dysfunction in a‐iCMs exposed to hypertrophic adipocytes. In the heart, metformin's activation of AMPK improves cardiac function by reducing inflammation and oxidative stress [46], enhancing fatty acid oxidation [47], protecting against insulin resistance [48], and mitigating fibrosis [49]. At the CM level, low‐dose metformin (<2.5 mM) enhances cellular respiration by stimulating mitochondrial biogenesis via AMPK signaling [50]. It also preserves mitochondrial membrane potential [51], reduces reactive oxygen species (ROS) production [51], and regulates autophagy [52] to reverse obesity‐related CM dysfunction. At the adipocyte level, metformin reduces circulating FFAs and adipose tissue lipolysis [53]. These effects are promising to collectively protect CMs from lipotoxicity, metabolic stress, and contractile dysfunction associated with obesity‐induced cardiac injury. Using 3D bioprinted constructs, we tested whether metformin treatment could mitigate hypertrophic adipocyte‐induced electrophysiological and metabolic impairments in a‐iCMs.

In this study, we established a human‐derived 3D bioprinted model that integrates ADSC‐derived hypertrophic adipocytes and a‐iCMs to replicate key features of obesity‐related cardiac dysfunction. We demonstrated that hypertrophic adipocytes disrupt a‐iCM metabolic function, contractility, calcium handling, and insulin signaling through both paracrine and/or juxtacrine interactions. Furthermore, we validated the therapeutic screening potential of constructs by demonstrating metformin's ability to modulate electrophysiological and metabolic impairments in 3D OAdi‐a‐iCMs. Engineered EAT‐myocardium models provide a biomimetic platform to investigate the mechanisms underlying obesity‐induced cardiac dysfunction and enable high‐throughput drug discovery to develop targeted treatments for CVD comorbidities.

## 2 Results and Discussion

### 2.1 3D Hypertrophic Adipocytes Display Hallmarks of Obesity

#### 2.1.1 Lipid Droplet Enlargement and Cytoskeletal Changes

**FIGURE 1:**
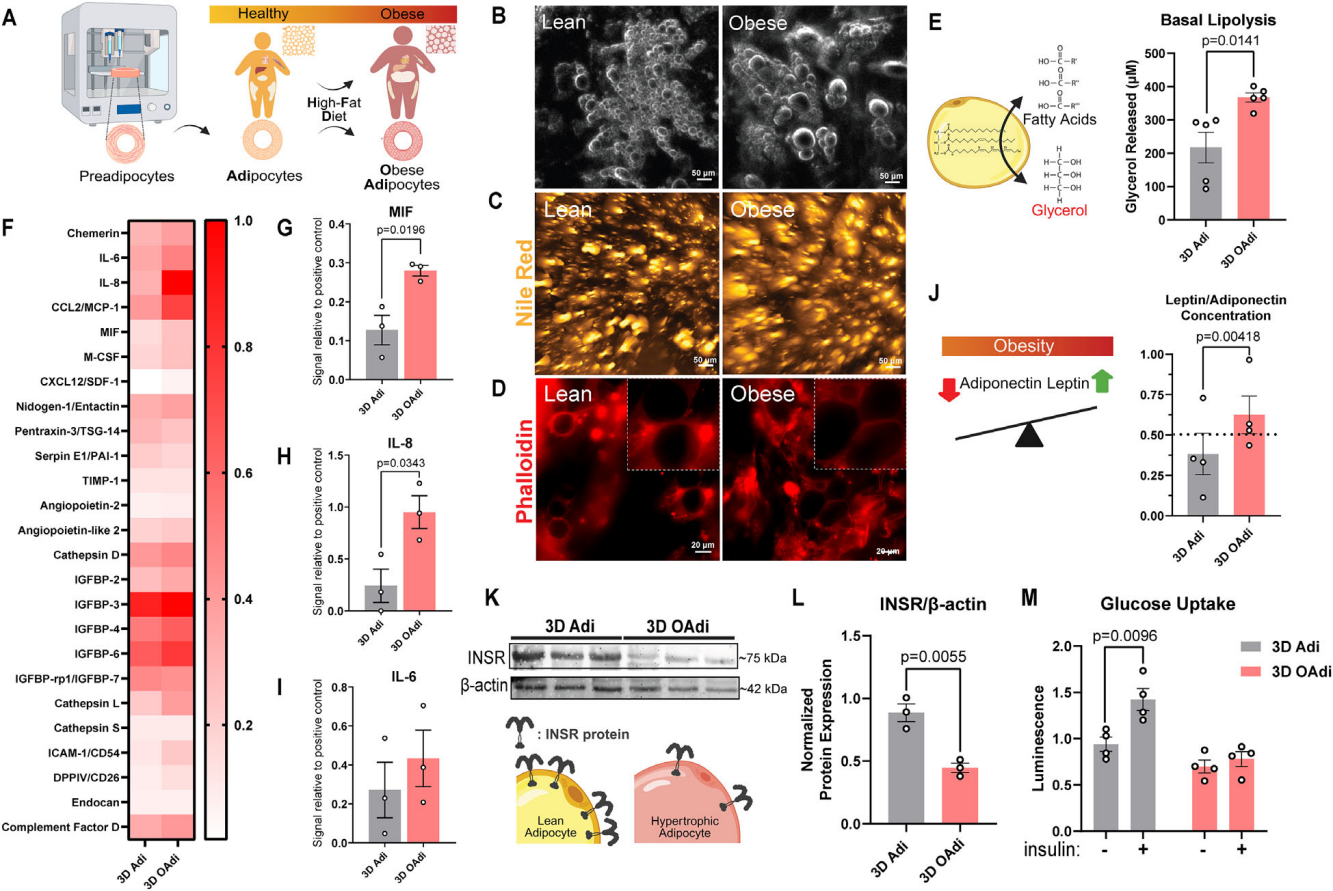
Characterization of 3D Adi and OAdi constructs according to well‐established hallmarks of obese adipocytes (A) Schematic displaying hypertrophy progression of obese adipocytes and recapitulation of this process using engineered constructs. (B) Bright‐field imaging (scale bar = 50 µm), (C) Nile Red staining (scale bar = 50 µm), and (D) Phalloidin (F‐actin) immunostaining (Red = F‐actin, (scale bar = 20 um)) of 3D Adi/OAdi. (E) Glycerol release per 3D Adi/OAdi construct over 48 h (n = 5, mean ± SEM; unpaired two‐tailed *t*‐test). (F) Human adipokine array: (G) MIF (n = 3, mean ± SEM; unpaired two‐tailed *t*‐test), (H) IL‐8 (n = 3, mean ± SEM; unpaired two‐tailed *t*‐test), (I) IL‐6 (n = 3, mean ± SEM; unpaired two‐tailed *t*‐test), and (J) Leptin/Adiponectin ratio quantification in 3D Adi/OAdi secretome (n = 4, mean ± SEM; batch‐adjusted linear regression). (K) INSR expression analyzed with western blot (n = 3) and (L) INSR/β‐actin protein expression in 3D Adi/OAdi constructs (n = 3, mean ± SEM; unpaired two‐tailed *t*‐test). (M) Basal and insulin‐dependent glucose uptake of the 3D Adi/OAdi (n = 4, mean ± SEM; unpaired two‐tailed *t*‐test).

In vivo, obese adipocytes are characterized by an increased capacity for triglyceride storage, leading to fewer but larger lipid droplets [54], which is indicative of a unilocular morphology typical of mature white adipocytes [55]. Previously, this phenomenon was replicated in vitro by exposing adipocytes to a fatty acid‐rich environment [16], which induces features similar to an in vivo obese phenotype. Here, we 3D bioprinted and differentiated ADSCs into adipocytes representing healthy and obese states through PA supplementation to induce adipocyte hypertrophy (Figure 1A). To assess lipid accumulation, we performed bright‐field (BF) imaging and Nile Red staining after a 6‐day PA treatment, revealing an increase in lipid droplet size in the obese constructs (3D OAdi) compared to controls (3D Adi). BF images demonstrated clear morphological distinctions, with 3D OAdi exhibiting larger lipid droplets compared to the control (Figure 1B, Figure S1B). Nile Red staining confirmed the accumulation of lipids, with 3D OAdi showing larger lipid droplet formation compared to the control (Figure 1C, Figure S1C,D). Quantification demonstrated a significant increase in lipid droplet size in 3D OAdi (*p*<0.0001, Figure S1E).

The organization of F‐actin around the 3D Adi and OAdi constructs was evaluated by F‐actin staining, revealing altered cortical actin structures in the obese constructs relative to the control, recapitulating the cytoskeletal changes driven by obesity‐induced ECM remodeling (Figure 1D). Cytoskeletal alterations observed in PA‐treated adipocytes reflect an adaptive response to accommodate lipid droplet enlargement in hypertrophic adipocytes. The adipocyte hypertrophy and alteration of actin cytoskeleton organization were reported to compromise adipocyte's ability to respond to insulin and effectively translocate glucose transporters, thereby impairing glucose uptake and contributing to the development of insulin resistance [56]. These results confirm that lipid droplet enlargement in 3D OAdi is accompanied by cytoskeletal alterations, supporting key structural hallmarks expected in obesity‐associated adipocyte dysfunction.

#### 2.1.2 Secretome Analysis and Cytokine Profiling

Lipolysis is the metabolic process by which triglycerides, stored in adipocytes, are broken down into FFA and glycerol. In a healthy heart, FFA secreted by cardiac fat depots supply over 60% of the energy needed for contractile function [5]. In lean adipocytes, the processes of energy uptake and release are tightly regulated, maintaining balanced lipolysis, where triglycerides are hydrolyzed into FFAs and glycerol as needed [57]. In contrast, hypertrophic adipocytes exhibit elevated basal lipolysis, resulting in an increased breakdown of triglycerides and FFA release [58]. When FFAs are chronically elevated, excess fatty acids accumulate in neighboring tissues, including the myocardium, which lacks the fat storage capacity to handle them efficiently [59]. We evaluated the basal lipolytic activity of the 3D Adi/OAdi by measuring glycerol release from the constructs as an indicator of lipolysis. 3D OAdi exhibited significantly increased basal lipolysis compared to 3D Adi (p = 0.0141, Figure 1E), as measured by elevated glycerol release. This finding aligns with in vivo dyslipidemic profile [60] and supports in vitro findings on 3D PA‐induced hypertrophic ADSC‐derived adipocytes [16].

Macrophage migration inhibitory factor (MIF), IL‐8, and IL‐6 are proinflammatory cytokines that play a key role in modulating immune responses, promoting macrophage retention, and contributing to chronic inflammation, which are all hallmarks of obesity‐related tissue dysfunction [61, 62, 63]. Human cytokine array analysis (Figure 1F) revealed significantly higher levels of MIF (p = 0.0196, Figure 1G) and IL‐8 (p = 0.0343, Figure 1H) and a substantial increase in IL‐6 (Figure 1I) in 3D OAdi compared to the lean control.

Leptin and adiponectin are adipokine hormones that play key roles in metabolic regulation [64]. Leptin normally suppresses appetite and promotes energy expenditure [65], while adiponectin enhances insulin sensitivity and has anti‐inflammatory properties [6]. Another consequence of adipocyte hypertrophy in obesity is the decrease in adiponectin secretion and a corresponding increase in leptin release, disrupting metabolic homeostasis and promoting insulin resistance and inflammation [66]. Under physiological conditions, the leptin‐to‐adiponectin ratio is typically below 0.5, and values exceeding this threshold have been associated with an increased risk of CVD [67]. 3D OAdi constructs showed a modest increase in leptin (Figure S1F) and a significant decrease in adiponectin (p = 0.03666, Figure S1G), resulting in a significant increase (p = 0.00418) in leptin‐to‐adiponectin ratio compared to the lean control and exceeded the 0.5 threshold (0.6243±0.2338, Figure 1J). Taken together, these findings indicate that 3D OAdi constructs effectively recapitulate obesity‐induced alterations in the adipose tissue secretome, including enhanced lipolysis, inflammatory cytokine secretion, and hormonal imbalance.

#### 2.1.3 Insulin Receptor Prevalence and Activation

Adipocytes express insulin receptors (INSR) that mediate glucose uptake, lipid metabolism, and energy storage in response to insulin signaling [68]. Obesity is the most common cause of insulin resistance, a condition in which insulin‐sensitive tissues, such as adipose tissue, skeletal muscle, and liver, fail to respond adequately to circulating insulin [69]. In individuals with obesity or diabetes, this impaired response is attributed to reduced surface expression of the insulin receptor (INSR) and diminished INSR kinase activity [70], leading to the development of type 2 diabetes mellitus (T2D) and CVD. INSR expression is decreased in the adipose tissue of obese patients compared to normal‐weight controls, with a progressive decline observed as BMI increases [69]. Similar to in vivo results, PA‐induced 3D OAdi had significantly reduced INSR expression compared to the lean control (p = 0.0055, Figure 1K,L). Notably, this reduction in INSR expression was observed in the absence of systemic inflammation or immune cell involvement, indicating that palmitate‐induced adipocyte hypertrophy alone is sufficient to impair insulin receptor expression in adipocytes.

To determine whether reduced INSR expression in 3D OAdi translates to impaired metabolic function, we assessed insulin‐stimulated glucose uptake as a functional readout of insulin responsiveness. While insulin treatment significantly increased glucose uptake in 3D Adi constructs (p  =  0.0096), 3D OAdi showed no significant response, indicating loss of insulin sensitivity in hypertrophic adipocytes (Figure 1M). Additionally, there was a substantial decrease in basal glucose uptake in 3D OAdi constructs compared to 3D Adi controls (p = 0.0577, Figure 1M). This could be an outcome of increased reliance on fatty acid oxidation due to HFD, or alternatively, a result of impaired basal glucose transporter expression and function.

Combined with elevated basal lipolysis, these findings could point to the presence of a vicious cycle, where elevated FFAs impair insulin signaling, and insulin resistance further promotes lipolysis [60]. Additionally, the increased leptin‐to‐adiponectin ratio, which has been proposed as a marker of insulin resistance [71], may be a contributor to the disrupted glucose metabolism and insulin insensitivity we observed in 3D OAdi. Our findings are consistent with prior in vitro (murine adipocyte) and in vivo studies using fatty acid‐treated adipocytes and short‐term HFD‐fed mice [15], which demonstrated that adipocyte hypertrophy alone, induced without immune cell infiltration or systemic inflammation, is sufficient to impair insulin receptor expression and disrupt insulin‐stimulated glucose uptake. These findings indicate that 3D OAdi constructs effectively model obesity‐induced changes in adipose tissue, including enhanced lipid storage, structural remodeling, increased proinflammatory signaling, disrupted insulin signaling, and insulin‐dependent glucose uptake, making them a biomimetic platform for studying obesity‐induced cardiac dysfunction.

Although we opted to use subcutaneous ADSCs for engineering of 3D Adi/OAdi for practical considerations, including their availability from elective procedures and suitability for high‐throughput fabrication and therapeutic screening, we additionally evaluated whether our bioprinting and differentiation workflows support engineering of cardiac fat–depot–specific adipocyte constructs. Subcutaneous ADSCs and human EAT stromal vascular fraction (SVF) cells were utilized to fabricate lean and obese adipocyte constructs derived from two different fat depots (3D s‐Adi/s‐OAdi, 3D e‐Adi/e‐OAdi), and their morphologies and secretome were characterized. Similar to previous in vivo findings, lean subcutaneous adipocytes contained larger lipid droplets than lean EAT adipocytes [72], while both depots displayed substantial lipid droplet enlargement following hypertrophy (Figure S2A). Moreover, both engineered fat depot's secretory profiles showed obesity‐associated adipokine changes, including increased leptin and decreased adiponectin, as well as elevated proinflammatory cytokines/chemokines such as IL‐6, IL‐8/CXCL8, MCP‐1/CCL2, and MIF (Figure S2B). These results confirm that both engineered fat depots (3D s‐OAdi, 3D e‐OAdi) recapitulate the morphology and adipokine profiles of adipocytes in obesity.

### 2.2 3D Hypertrophic Adipocyte Secretome Induces Metabolic Dysfunction in Atrial Cardiomyocytes

#### 2.2.1 Mitochondrial Bioenergetic Function

**FIGURE 2:**
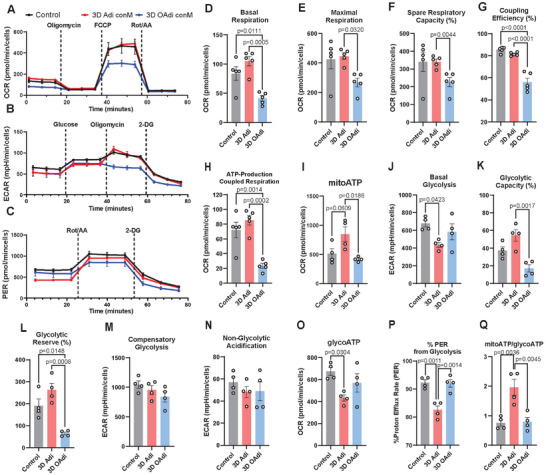
Metabolic profiling of a‐iCMs exposed to 3D Adi/OAdi ConM reveals impaired mitochondrial function and altered glycolytic activity. (A) Oxygen consumption rate (OCR) following sequential addition of oligomycin, FCCP, and rot/AA (Seahorse Mito Stress test) (n = 5). (B) Extracellular acidification rate (ECAR) following glucose, oligomycin, and 2‐DG injections (Seahorse Glycolysis Stress test) (n = 4). (C) Proton efflux rate (PER) analysis rot/AA, and 2‐DG injections (Seahorse Glycolytic Rate test) (n = 4). Quantification of (D) basal respiration (n = 5), (E) maximal respiration (n = 5), (F) spare respiratory capacity (n = 5), (G) coupling efficacy, (H) ATP‐linked respiration (n = 5), (I) mitochondrial ATP production (mitoATP) (n = 4) (J) basal glycolysis (n = 4), (K) glycolytic capacity (n = 4), (L) glycolytic reserve (n = 4), (M) compensatory glycolysis (n = 4), (N) non‐glycolytic acidification (n = 4), (O) glycolysis‐dependent ATP production (glycoATP) (n = 4), and (P) percent PER from glycolysis (n = 4) (Q) The mitoATP/glycoATP ratio of control (RPMI 1640 supplemented with 2% B27 Supplement (50X), minus antioxidants) and ConM (3D Adi/OAdi) treated a‐iCMs (n = 4). Data was represented as mean ± SEM and one‐way ANOVA with Tukey's post hoc test was used for all analysis in the panel.

Obese patients have alterations in myocardial substrate utilization [73] and reduced ATP production/utilization efficiency [74], indicating mitochondrial dysfunction. Earlier studies suggested that CM mitochondrial dysfunction is caused by myocardial lipid accumulation, impaired glucose tolerance, and insulin resistance [75]. Here, we evaluated and compared the paracrine effects of 3D Adi and 3D OAdi on the mitochondrial function of a‐iCMs using Seahorse extracellular flux analysis (Figure 2A). We observed that 3D OAdi conditioned media (ConM) treated a‐iCMs exhibited significantly reduced basal respiration compared to a‐iCMs cultured with either the control (p = 0.0111) or 3D Adi secretome (p = 0.0005, Figure 2D). Moreover, a‐iCMs cultured with the 3D OAdi ConM demonstrated a significant reduction in maximal respiration (p = 0.0320, Figure 2E) and spare respiratory capacity (p = 0.0044, Figure 2F) compared to 3D Adi group, which indicates a diminished ability of the mitochondria to adapt to increased energy demands during periods of stress. Coupling efficiency, which reflects how effectively mitochondria convert electron transport chain activity into ATP production, was also significantly reduced in a‐iCMs cultured with the 3D OAdi ConM, both compared to the control and 3D Adi group (*p*<0.0001, Figure 2G). Furthermore, ATP‐production coupled respiration was significantly decreased in a‐iCMs cultured with the 3D OAdi ConM, both compared to the control (p = 0.0014) and 3D Adi group (p = 0.0002, Figure 2H). Mitochondrial ATP production (mitoATP), reflecting the ATP generated through mitochondrial oxidative phosphorylation (OXPHOS), was significantly lower in 3D OAdi ConM‐treated a‐iCMs compared to 3D Adi (p = 0.0186, Figure 2I). Importantly, mitoATP of a‐iCM treated with 3D Adi was substantially increased compared to control, indicating secreted factors from healthy adipocytes may enhance mitochondrial OXPHOS and support cardiomyocyte energy metabolism. Taken together, these findings suggest that the secretome of hypertrophic adipocytes impairs mitochondrial function of a‐iCMs through paracrine mechanisms, evidenced by reduced basal and maximal respiration, spare capacity, coupling efficiency, and mitochondrial ATP production. In contrast, secreted factors from lean adipocytes promote a metabolic shift from glycolysis toward OXPHOS, potentially priming a‐iCMs toward a more mature energetic phenotype seen in vivo [76].

To further validate these mitochondrial impairments, we assessed mitochondrial membrane potential using JC‐1 staining (Figure S3A). JC‐1 exhibits a membrane potential‐dependent shift in fluorescence emission, with increased red fluorescence indicating more highly polarized and metabolically active mitochondria. a‐iCMs treated with 3D OAdi ConM exhibited a significant decrease in the JC‐1 red/green ratio (p = 0.0252, Figure S3D), indicating decreased mitochondrial polarization. We next examined oxidative stress, which parallels the decrease in JC‐1 ratio, using CellROX (Figure S3B). a‐iCMs exposed to 3D OAdi ConM demonstrated a substantial increase in oxidative stress relative to control (p = 0.0681, Figure S3E). This elevation in ROS production could indicate that hypertrophic adipocyte secretome disrupts mitochondrial redox homeostasis and contributes to membrane depolarization. Together, the JC‐1 and ROS analyses corroborate the Seahorse measurements, demonstrating that factors secreted by hypertrophic adipocytes drive mitochondrial dysfunction and oxidative stress in a‐iCMs, whereas the lean adipocyte secretome supports oxidative metabolism without altering the already high mitochondrial polarization of healthy a‐iCMs.

#### 2.2.2 Glycolytic Function

When CMs sustain mitochondrial damage, glycolysis is expected to increase as the impaired mitochondria are unable to generate adequate ATP through OXPHOS [77]. This metabolic flexibility forces the cell to rely on the less efficient but faster glycolytic pathway to meet its energy demands and sustain cellular functions [77]. However, obesity‐related cardiometabolic diseases are characterized by metabolic inflexibility [78], where cells are unable to switch between fuel sources such as glucose and fatty acids. Here, we compare the paracrine effects of 3D Adi and OAdi on the glycolytic function of a‐iCMs using Seahorse extracellular flux analysis (Figure 2B,C). The basal glycolysis of 3D Adi‐ConM‐treated a‐iCMs was significantly lower compared to the control (Figure 2J, p = 0.0423). Combined with the Mito Stress assay results (Figure 2A‐I), the decrease in basal glycolysis might reflect an early metabolic shift from glycolysis toward OXPHOS induced by lean adipocyte secreted factors. Glycolytic capacity, which measures the maximum glycolytic potential, was significantly reduced in the 3D OAdi ConM‐treated group compared to 3D Adi ConM‐treated group (Figure 2K, p = 0.0017). Glycolytic reserve, representing the additional glycolytic capacity available under stress, was significantly impaired in 3D OAdi ConM‐treated a‐iCMs compared to the control (p = 0.0148) and 3D Adi ConM‐treated group (p = 0.0008, Figure 2L). Notably, there was a substantial increase in glycolytic capacity and reserve in a‐iCMs treated with 3D Adi ConM compared to control (Figure 2K,L), suggesting that while mitochondrial ATP production is enhanced, the capacity for glycolytic compensation is also preserved, and even markedly increased, indicating improved metabolic flexibility.

There was a marked decrease in compensatory glycolysis, which shows reduced ability of CM to shift toward ATP production from glycolysis (glycoATP) when mitochondrial function is impaired in the OAdi group compared to control (Figure 2M). Non‐glycolytic acidification, which reflects proton production from sources other than glycolysis, remained similar across the groups (Figure 2N). This suggests that the observed differences in extracellular acidification were primarily driven by changes in glycolytic activity, rather than alterations in fatty acid oxidation or other non‐glycolytic metabolic pathways. glycoATP was significantly lower in the 3D Adi CM‐treated a‐iCMs compared to the other groups (Figure 2O, p = 0.0304). The percentage of proton efflux rate (PER) derived from glycolysis was significantly reduced in the 3D Adi ConM group compared to the control (p = 0.0011) and 3D OAdi group (p = 0.0014, Figure 2P), which further indicated a diminished reliance on glycolysis for extracellular acidification in 3D Adi group. The ratio of mitoATP to glycoATP production was significantly higher in the 3D Adi group compared to the control (p = 0.0036) and 3D OAdi (Figure 2Q, p = 0.0045), confirming a metabolic shift toward mitochondrial oxidative phosphorylation induced by lean adipocyte secretome. Taken together, 3D OAdi ConM‐treated a‐iCMs exhibit marked metabolic inflexibility, characterized by an impaired ability to upregulate glycolysis in response to mitochondrial dysfunction, suggesting that paracrine signals from hypertrophic adipocytes compromise their capacity to adapt to energetic stress. In contrast, 3D Adi ConM‐treated a‐iCMs displayed enhanced metabolic flexibility, characterized by increased mitochondrial ATP production alongside preserved and elevated glycolytic reserve, suggesting that lean adipocyte secretome supports a balanced and adaptive energy metabolism in a‐iCMs.

In our supplementary studies, we also included an expanded analysis with additional control groups (Figure S4), such as two different concentrations of adiponectin and leptin correlating the 3D Adi (adiponectin‐high, leptin‐low) and OAdi secretome content (adiponectin‐low/leptin‐high, as well as two different concentrations of PA to reflect the dose‐dependent metabolic effects of lipotoxicity. 3D OAdi ConM and Leptin‐high treated a‐iCMs had significantly lower spare respiratory capacity compared to 3D Adi ConM, indicating the leptin content of 3D OAdi ConM might be contributing to a reduced ability to meet increased energy demands under stress (Figure S3D). a‐iCMs treated with increasing concentrations of leptin exhibited increased glycoATP observed at the highest leptin dose compared to lower concentrations (Figure S4F), consistent with in vivo studies showing leptin enhances glucose utilization [79]. Coupling efficacy and mitoATP production of 3D OAdi ConM treated a‐iCMs were significantly reduced (Figure S4E, G) compared to every other group, which suggests that the changes seen in mitochondrial function of 3D OAdi ConM‐treated a‐iCMs might be due to some other small molecule or a combinatorial effect of the contents of this secretome.

Although glycolysis was relatively similar across groups (Figure S4I), PA‐treated group exhibited a similar reduction in glycolytic capacity and reserve (%) as observed in a‐iCMs exposed to 3D OAdi ConM (Figure S4J‐K), suggesting that the elevated fat content in the 3D OAdi secretome may contribute to impaired glycolytic function in a‐iCMs. Lastly, non‐glycolytic acidification, typically linked to other cellular or metabolic processes that generate protons, was significantly elevated in the PA‐high group, potentially reflecting increased fatty acid oxidation (Figure S4L). Together, these findings demonstrate that the secretome of hypertrophic adipocytes induces profound metabolic dysfunction in a‐iCM by impairing both mitochondrial OXPHOS and glycolytic adaptability, whereas the lean adipocyte secretome promotes metabolic flexibility and a shift toward a more efficient, OXPHOS‐dominant energetic phenotype of a‐iCMs similar to healthy adult human CM [80].

Despite the limitations of 2D adipocyte cultures (e.g., detachment from the tissue culture plate during cultivation periods longer than 28 days [81], dedifferentiation after only one week of culture [82]), we also ran supplementary experiments (Figures S6 and S7) where we collected ConM from both 2D adipocytes (2D Adi) and hypertrophic adipocytes (2D OAdi) and applied it to a‐iCMs. We observed decreased mitochondrial function in both groups (Figure S7A‐D), with the hypertrophic adipocyte group showing even lower glycoATP levels (Figure S7E). Overall, culturing adipocytes as monolayers (2D Adi/OAdi) did not yield a clear distinction in their paracrine effects on a‐iCMs. We suspect this may be due to adipocytes, even lean ones, adopting a pathological phenotype when maintained on plastic surfaces, thereby impairing both mitochondrial and glycolytic function of a‐iCMs, which warrants further investigation.

### 2.3 3D Hypertrophic Adipocytes Secretome Alters Cardiac Function of 2D a‐iCMs

#### 2.3.1 Beating Kinetics

**FIGURE 3:**
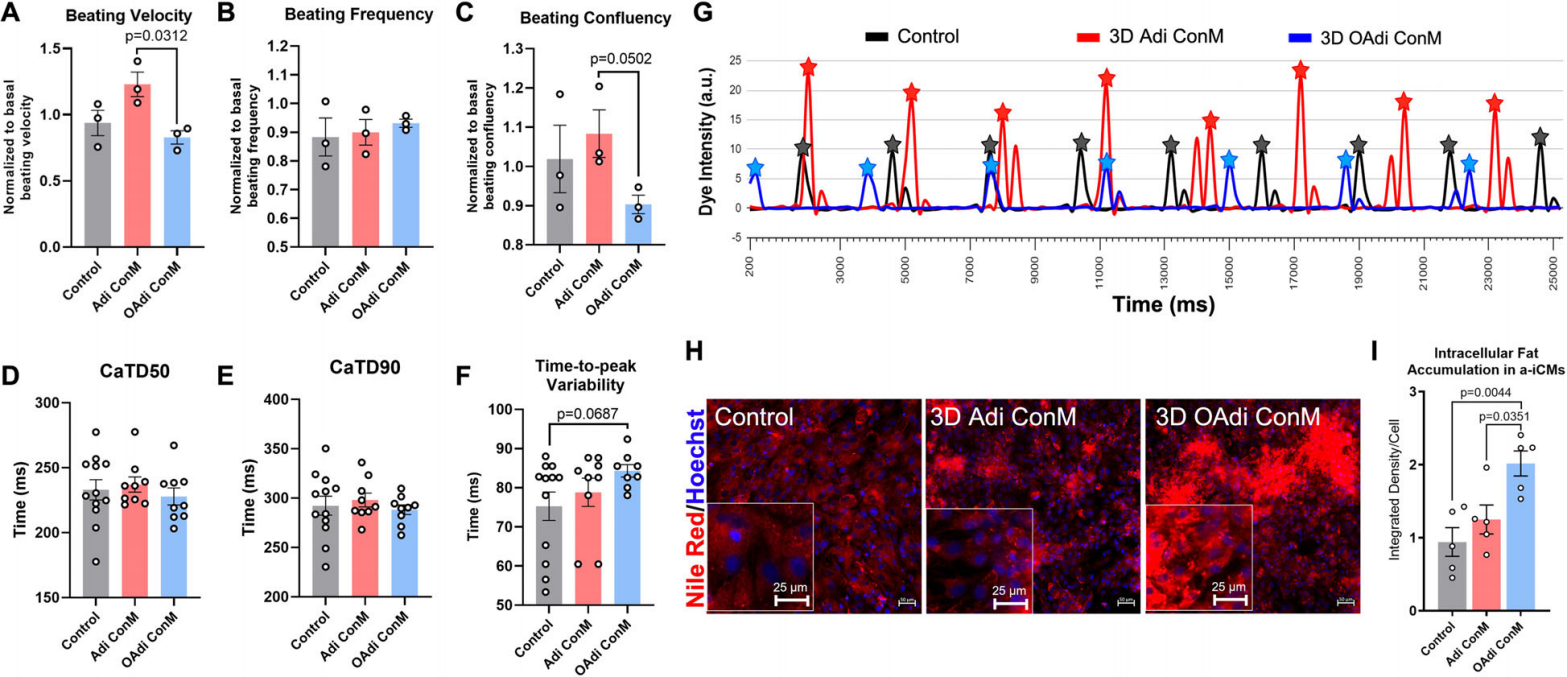
Alteration of beating characteristics in a‐iCMs exposed to 3D OAdi conditioned media (ConM) and ectopic fat accumulation. Brightfield beating analysis of (A) beating velocity (n = 3, mean ± SEM; One‐way ANOVA with Tukey's post hoc test), (B) beating frequency (n = 3), and (C) beating confluency (n = 3, mean ± SEM; One‐way ANOVA with Tukey's post hoc test), calcium flux analysis of (D) CaTD50 (n = 9‐12), (E), CaTD90 (n = 9‐12), (F) time‐to‐peak variability in a‐iCMs treated with ConM (n = 8‐12, mean ± SEM; One‐way ANOVA with Tukey's post hoc test), (G) representative calcium transient waveforms (fluo‐4 dye intensity (a.u.) is plotted over time (ms), selected calcium transient peaks used for downstream quantitative analyses are indicated by stars), (H) nile Red staining (red: Nile Red, lipid, blue: Hoechst33342, nuclei) (scale bar = 50 um) and magnified image (scale bar = 25 um) and (I) quantification of integrated density of ectopic lipid accumulation/cell of control (RPMI 1640 supplemented with 2% B27 Supplement (50X), minus antioxidants) and ConM (3D Adi/OAdi) treated a‐iCMs (n = 4) (n = 5, mean ± SEM; One‐way ANOVA with Tukey's post hoc test).

Due to the altered metabolic function of the myocardium, the obese heart is in an energetically disadvantaged position [83]. Since metabolic function is directly and critically related to the beating of CMs [84], failing human CMs exhibit reduced contractility [85]. We recorded the spontaneous beating of the a‐iCMs treated with 3D Adi/OAdi ConM and visualized the beating kinetics using vector heat maps to quantify alterations in the contractile dynamics of a‐iCMs. The beating velocity of the 3D OAdi ConM‐treated a‐iCMs was significantly decreased compared to the 3D Adi ConM group (p = 0.0312, Figure 3A), whereas beating frequency between groups was not significantly altered (Figure 3B). Beating confluency was substantially decreased in 3D OAdi ConM‐treated a‐iCMs compared to 3D Adi ConM‐treated a‐iCMs (Figure 3C). In supplementary studies, we once again included leptin low/high and adiponectin low/high groups to characterize the effect of major adipokines on the beating characteristics of a‐iCMs (Figure S8). The beating velocity of 3D OAdi ConM‐treated a‐iCMs was significantly reduced compared to both adiponectin‐containing groups (p = 0.0106 and p = 0.0070), in addition to the 3D Adi ConM group (Figure S8A). Additionally, leptin‐containing groups had a similar decrease with 3D OAdi group in terms of beating velocity compared to 3D Adi ConM and adiponectin‐containing groups (Figure S8A), indicating that leptin and adiponectin components released by adipocytes might be players in alterations in contractile function of a‐iCMs in obesity. Beating frequencies remained relatively consistent across all groups (Figure S8B), and neither adiponectin nor leptin supplementation resulted in significant differences in beating confluency (Figure S8C), suggesting that while these adipokines influence contractile velocity, they may not substantially affect beating confluency under the tested conditions.

#### 2.3.2 Calcium Transient

Analysis of calcium transients is considered complementary to the beating kinetics of CMs, as calcium ions enter the cells during each beat and contribute to the electrical signal [86]. We recorded the calcium transient during spontaneous beating (Movie S2) and quantified CaTD50 (calcium transient duration at 50% decay), CaTD90 (calcium transient duration at 90% decay), and time to peak (TTP) variability. While CaTD50 and CaTD90 remained relatively consistent (Figure 3D,E), TTP variability increased substantially in the a‐iCMs treated with 3D OAdi ConM compared to the control (p = 0.0687, Figure 3F), indicating potential disturbances in contraction timing or calcium handling in a‐iCMs treated with 3D OAdi ConM. Additionally, we observed peak intensity increase in a‐iCMs treated with 3D Adi ConM (Figure 3G). Taken together, results suggest that factors secreted by 3D Adi may enhance cardiac function of a‐iCM by supporting metabolic and contractile function.

### 2.4 3D Hypertrophic Adipocytes Secretome Induces Intracellular Fat Accumulation in a‐iCMs

Under physiological conditions, CMs store a small amount of intracellular triglycerides within lipid droplets, serving as an energy reserve to meet the high ATP demand of the heart, primarily through fatty acid oxidation. This finely tuned balance between lipid uptake, storage, and oxidation preserves cardiac metabolic flexibility [87]. However, in obesity, excess circulating FFA disrupts this balance, leading to ectopic fat accumulation within the myocardium [88]. Consequently, surplus lipids not only increase myocardial triglyceride content but also elevate levels of lipotoxic intermediates such as diacylglycerols and ceramides [89], leading to increased mitochondrial and endoplasmic reticulum (ER) stress, and trigger proinflammatory and proapoptotic pathways [90]. Nile Red staining revealed minimal lipid accumulation in control a‐iCMs, a modest increase in cells exposed to 3D Adi ConM, and a significant increase in intracellular lipid deposition in a‐iCMs treated with 3D OAdi ConM (Figure 3H). Quantification confirmed significantly higher integrated lipid density per cell in the OAdi ConM group compared to both control (p = 0.0044) and Adi ConM groups (p = 0.0351, Figure 3I). These findings suggest that the 3D OAdi ConM, enriched in fatty acids, drives ectopic fat accumulation in monolayer a‐iCMs, which may be driving ROS production (Figure S3B), impaired contractility (Figure 3A), mitochondrial and glycolytic dysfunction, and metabolic inflexibility (Figure 2). Taken together, these results indicate that lean adipocytes support cardiac function while the detrimental effects, such as ectopic fat accumulation and mitochondrial impairments, arise from adipocyte dysfunction, underscoring the importance of adipocyte phenotype in modulating myocardial physiology.

### 2.5 Engineering and Characterizing the 3D Fat‐Myocardium Model

#### 2.5.1 Cell Colocalization and Viability of the Model

**FIGURE 4:**
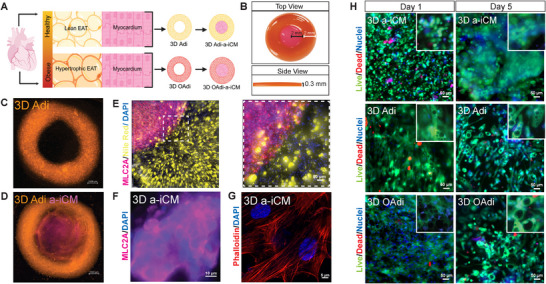
3D bioprinting of Adi/OAdi a‐iCM constructs. (A) Schematic representation of lean and hypertrophic EAT and the process of 3D bioprinting of Adi/OAdi a‐iCM constructs. (B) Image of the 3D bioprinted concentric ring construct (4 mm outer diameter, 2 mm inner diameter, 0.3 mm height). Cell colocalization in the constructs using CellTracker. (C) 3D Adipocytes: Orange, Scale bar: 1000 µm) (D) 3D Adipocytes: Orange, 3D a‐iCMs: Far Red (Scale bar: 1000 µm), post‐printing. (E) Immunofluorescence images of a‐iCM marker (MLC2A, magenta) and adipocyte marker (Nile Red, yellow) (Scale bar: 50 µm, Magnified Image Scale Bar: 100 µm) at Day 1 of co‐culture (F) MLC2A/DAPI staining confirming atrial iCM identity (Scale bar: 10 µm). (G) Phalloidin/DAPI staining visualizing actin cytoskeleton organization in 3D a‐iCMs (Scale bar: 5 µm) at Day 5 of co‐culture. (H) Live‐Dead imaging of 3D construct regions at Days 1 and 5 of co‐culture (Green: live cells, red: dead cells, blue: nuclei (Hoechst 33342), Scale bar: 50 µm).

After determining the direct paracrine effects of 3D Adi/OAdi ConM on a‐iCMs, we constructed a fully 3D model (Figure 4A) to more accurately recapitulate the spatial and cellular interactions between adipocytes and CMs, enabling evaluation of both paracrine and juxtacrine mechanisms in a physiologically relevant microenvironment. The concentric circle model (Figure 4B) was designed to mimic the anatomical arrangement where EAT lies directly adjacent to the myocardium, sharing the same microcirculation and enabling direct cell‐cell and paracrine interactions. To colocalize the cells, 3D Adi and OAdi constructs were tagged with Orange CellTracker (Figure 4C). a‐iCMs were tagged with Far Red CellTracker, incorporated into the GelMA‐collagen hybrid bioink as explained in Section 4.4. and 3D bioprinted into the middle of the 3D Adi and OAdi constructs (Figure 4D). In parallel studies, constructs were stained using cell‐specific markers, MLC2A (atrial CM specific, Figure 4E,F) and Nile Red (lipid specific, Figure 4E). Additionally, phalloidin localization in the 3D a‐iCMs after 5 days in culture revealed well‐organized cytoskeletal architecture and aligned CMs, indicating a‐iCMs established structural integrity after 3D bioprinting (Figure 4G). 24 h after a‐iCM bioprinting, cell viability was assessed with Live/Dead staining. All 3D bioprinted groups (3D Adi/OAdi (Figure S9A), and 3D a‐iCM) had high viability (>93%) (Figure 4H, Figure S9B). By Day 5, the a‐iCMs organized into clusters, tissue‐like structures, while maintaining high viability across all groups (Figure 4H, Figure S9C).

### 2.6 Effect of 3D Hypertrophic Adipocytes on Beating Activity and Gap Junction Expression of 3D a‐iCMs

#### 2.6.1 3D Hypertrophic Adipocytes Alter Cardiac Function of 3D a‐iCMs

##### 2.6.1.1 Beating Kinetics

**FIGURE 5:**
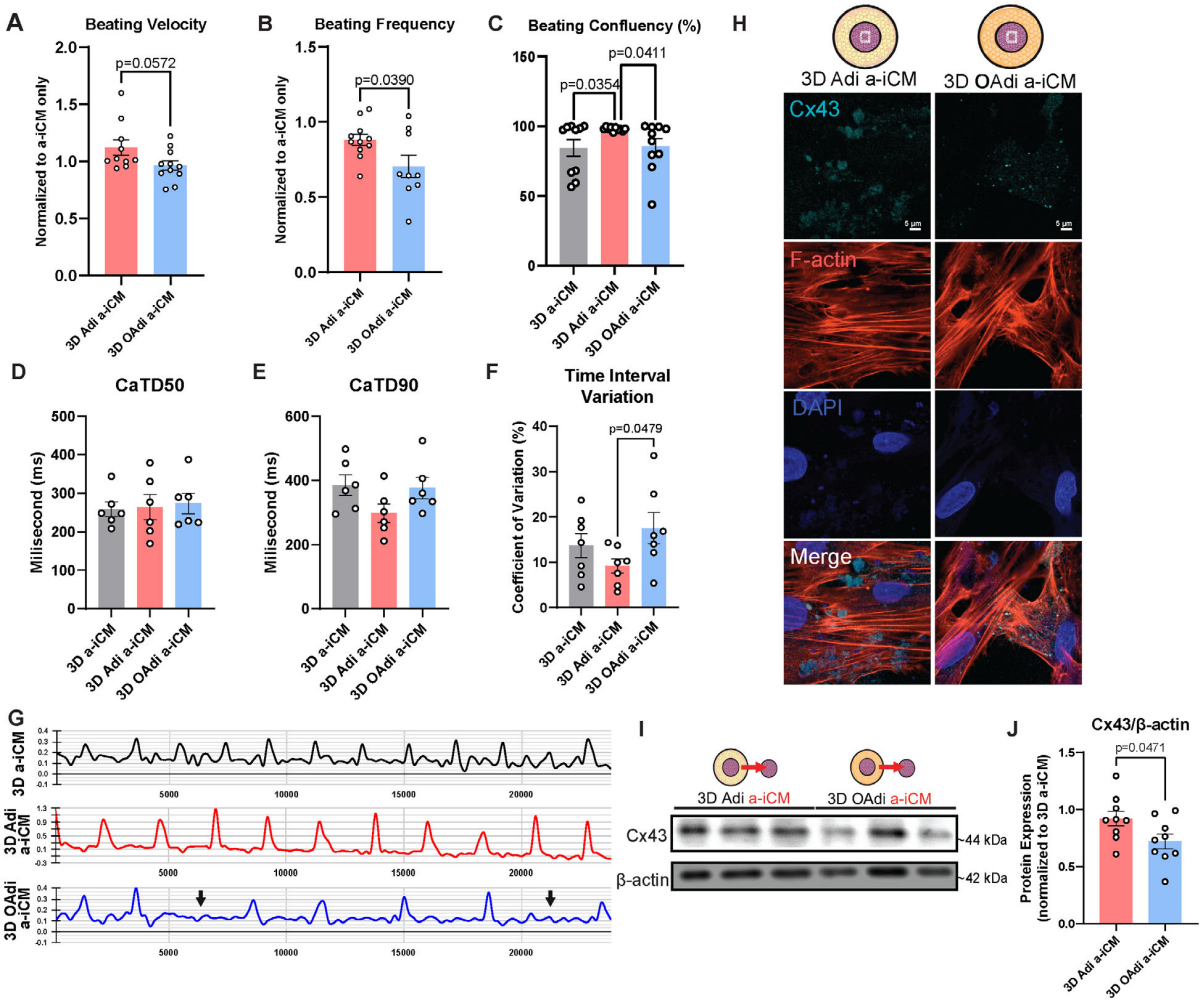
OAdi a‐iCM constructs exhibit decreased beating frequency, increased time interval variation, and disrupted gap junction expression. (A) Beating velocity (n = 11, mean ± SEM; unpaired two‐tailed *t*‐test), (B) beating frequency (n = 9‐11, mean ± SEM; unpaired two‐tailed *t*‐test), and (C) beating confluency (n = 10, mean ± SEM; One‐way ANOVA with Tukey's post hoc test), (D) CaTD50 with beat‐rate correction (n = 7, mean ± SEM), (E), CaTD90 with beat‐rate correction (n = 6, mean ± SEM; One‐way ANOVA with Tukey's post hoc test), (F) time‐to‐peak variability (n = 7, mean ± SEM; One‐way ANOVA with Tukey's post hoc test), (G) representative calcium transient waveforms, (H) immunofluorescence staining of gap junction Connexin43 (Cx43) and F‐actin (Cx43: cyan, F‐actin: red, and DAPI: blue) (Scale bar: 5 µm), (I) Western blot analysis and (J) quantification of Cx43 normalized to β‐actin (n = 9, mean ± SEM; unpaired two‐tailed *t*‐test) a‐iCMs cultured either alone, with 3D Adi, or 3D OAdi for five days.

Beyond paracrine signaling, the direct physical interaction between adipocytes and CMs plays a critical role in influencing cardiac function of CMs [40]. We captured the spontaneous beating of the 3D a‐iCMs cultured alone or with 3D Adi/OAdi cultures (Movie S1) and visualized their kinetics using vector heat maps. The maps revealed that a‐iCMs cultured with 3D Adi have tissue‐like beating with high confluency (Figure S10A), in contrast to a‐iCMs cultured with 3D OAdi or alone. a‐iCMs cultured with 3D OAdi exhibited a substantial decrease in beating velocity (Figure 5A) and a significant decrease in beating frequency compared to 3D Adi group (p = 0.0390, Figure 5B). The significant decrease in beating velocity observed in a‐iCM cocultured with 3D OAdi or a‐iCM treated with 3D OAdi ConM aligns with prior findings in which rat CMs exposed to human white adipocyte‐ConM exhibited an acute reduction in contraction amplitude [91]. In addition, a‐iCMs cultured with 3D Adi had significantly higher beating confluency with minimal variability compared to a‐iCMs cultured with 3D OAdi (p = 0.0411) or a‐iCM control (p = 0.0354, Figure 5C). Irregular, skipped beats were observed in 3D OAdi a‐iCMs as shown in Movies S3 and beating irregularity were subsequently quantified by interbeat interval (IBI) variability analysis from calcium flux data (described in the Calcium Transients section).

Compared to the ConM treatment, where a‐iCMs exposed to 3D OAdi ConM showed reduced beating velocity without significant changes in beating frequency, the 3D coculture model resulted in more pronounced impairments. a‐iCMs cocultured with 3D OAdi exhibited a marked decrease in beating velocity, a significant reduction in beating frequency, and significantly lower beating confluency compared to 3D Adi controls (Figure 5A–C). These findings suggest that direct physical interaction with 3D OAdi may exacerbate adipocyte hypertrophy‐associated changes in cardiac function of a‐iCMs relative to the 3D Adi group beyond the effects of secreted factors alone, emphasizing the importance of cell‐cell and cell‐matrix interactions in mimicking obesity‐induced cardiac dysfunction.

##### 2.6.1.2 Calcium Transient

In obesity, ectopic lipid accumulation in myocardium impairs mitochondria and the ER, which can disrupt calcium handling and promote arrhythmias via early/delayed afterdepolarizations [23]. To investigate how these mechanisms manifest in our models, the calcium transient during spontaneous beating of 3D bioprinted a‐iCMs, Adi a‐iCMs, and OAdi a‐iCMs was recorded. CaTD50, CaTD90, time‐to‐peak, and time interval variation were analyzed. CaTD50 and CaTD90 remained relatively unaffected across models (Figure 5D,E), which was correlated to in vivo findings in ovine model, where short‐term HFD exposure to ovine left atrial CMs had no significant impact on APD50 and late repolarization [21]. IBI variability, reflects the electrical stability and rhythmicity of CMs, and increased variability is often linked to arrhythmias [92, 93]. Increased IBI variability could indicate disruptions in electrical conduction, and triggered activity, commonly seen in obese patients [94]. These abnormalities increase the risk of asynchronous contractions and reentry circuits, key mechanisms of arrhythmogenesis [95]. 3D OAdi a‐iCMs showed significantly higher time interval variation compared to the Adi 3D a‐iCM (p = 0.0479, Figure 5F). Calcium transient waves of 3D a‐iCMs and 3D Adi a‐iCMs were regular, indicating consistent calcium cycling (Figure 5G) with regular and coordinated beating patterns. In contrast, 3D OAdi a‐iCMs demonstrated irregular transients with skipped beats (indicated by arrows) with increased time interval variation, similar to the BF video analysis (Figure 5G, Movie S4). This is further supported by the overlaid calcium transient peaks (Figure S10C), where the 3D Adi a‐iCM model exhibits sharper and more synchronized peaks compared to the broader and inconsistent peaks in the 3D OAdi a‐iCM model. Correlated to our results, studies in obese sheep have demonstrated EAT expansion leads to conduction abnormalities and a higher risk of atrial arrhythmias [96]. However, beating irregularities such as AF rarely occur naturally in animals and usually requires external induction, making it challenging to study its spontaneous onset and progression as in humans [97]. Therefore, our findings demonstrate that the 3D OAdi a‐iCM model effectively mimics obesity‐induced beating irregularity, offering a physiologically relevant platform to study beating irregularity in the absence of external induction. These findings suggest that lean, 3D Adi created a supportive microenvironment that maintains/enhances a‐iCMs calcium handling, rhythmicity, and synchronization, while the pathological effects of hypertrophic, 3D OAdi impair these critical functions shown here with significantly decreased beating frequency compared to 3D a‐iCM control or 3D Adi a‐iCMs, as well as increased IBI compared to 3D Adi a‐iCMs.

#### 2.6.2 3D Hypertrophic Adipocytes Alter Gap Junction Expression of a‐iCM

Gap junction channels are essential for electrical conduction and synchronized contraction by enabling ion and small molecule exchange between myocardial cells [98]. Connexin43 (Cx43) is the predominant cardiac gap junction protein responsible for facilitating direct electrical and metabolic coupling between CMs, ensuring synchronous impulse propagation and coordinated contraction [99]. Remodeling, downregulation, or dephosphorylation of Cx43 has been strongly associated with impaired electrical conduction, increased arrhythmic susceptibility, and heart failure in both animal models [100] and human cardiac disease [101]. Given the observed disturbances in calcium handling and rhythmicity in the 3D OAdi a‐iCM model, we next investigated the gap junction prevalence.

3D OAdi a‐iCMs exhibited marked decrease in Cx43 expression compared to 3D Adi a‐iCMs (Figure 5H). Cx43 protein expression obtained by western blot confirms this observation, with total Cx43 expression significantly lower in the 3D OAdi a‐iCMs compared to 3D Adi a‐iCMs (p = 0.0471, Figure 5I,J). The reduction in Cx43 abundance may contribute to the beating irregularities observed in the 3D OAdi a‐iCMs. These findings highlight that hypertrophic adipocytes not only disrupt CM function through metabolic and paracrine effects but might also be impairing electrical coupling by downregulating critical gap junction components. This provides an important mechanistic insight into how obesity‐driven adipose dysfunction may increase arrhythmic risk and promote cardiac dysfunction, underscoring the value of the 3D OAdi‐a‐iCM model for studying obesity‐related CVD.

### 2.7 3D Hypertrophic Adipocytes Decrease Insulin Receptor Phosphorylation and Glucose Uptake of a‐iCMs

**FIGURE 6:**
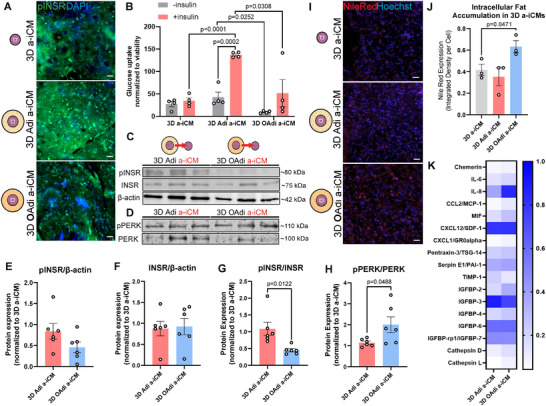
3D OAdi impairs glucose uptake and insulin signaling, and induces endoplasmic reticulum stress in a‐iCMs. (A) Representative immunofluorescence images of phosphorylated insulin receptor (green: pINSR) and nuclei (blue:DAPI) of the constructs (scale bar = 50 µm). (B) Basal and insulin‐dependent glucose uptake assay of 3D a‐iCM portion of constructs (n = 4, mean ± SEM; One‐way ANOVA with Tukey's post hoc test) (C) Western blot analysis of pINSR, INSR, and (D) pPERK, PERK in 3D a‐iCMs cultured with Adi/OAdi. Quantification of (E) pINSR/β‐actin (n = 6, mean ± SEM; unpaired two‐tailed *t*‐test) (F) INSR/β‐actin (n = 6, mean ± SEM; unpaired two‐tailed *t*‐test) (G) pINSR/INSR (n = 6, mean ± SEM; unpaired two‐tailed *t*‐test) (H) pPERK/PERK (n = 6, mean ± SEM; unpaired two‐tailed *t*‐test) (I) Representative lipid (red:Nile Red) and nuclei staining (blue:Hoechst33342) showing intracellular lipid accumulation in constructs (scale bar = 50 µm). (J) Quantification of Nile Red integrated intensity per cell (n = 4, mean ± SEM; One‐way ANOVA with Tukey's post hoc test). (K) Cytokine analysis of 3D Adi/OAdi a‐iCMs.

INSR are abundant in the heart and vasculature, regulating cardiac growth, survival, metabolism, and stress responses [102]. While insulin signaling adapts to metabolic changes to protect the heart from cardiotoxicity, its dysregulation in obesity and diabetes may contribute to myocardial dysfunction and heart failure progression [103]. In the atrium, insulin resistance induces atrial structural remodeling and disrupts intracellular calcium homeostasis, increasing susceptibility to arrhythmias [104]. It also induces mitochondrial dysfunction, ER stress and the unfolded protein response, oxidative stress, and inflammation [105]. To investigate the effects of hypertrophic adipocytes on INSR prevalence and activation, we assessed INSR and phosphorylated INSR (pINSR) expression in constructs. While INSR/β‐actin expressions of 3D Adi a‐iCMs and 3D OAdi a‐iCMs remained relatively similar (Figure 6C,F), pINSR/β‐actin expression of 3D Adi a‐iCMs was substantially higher than 3D OAdi a‐iCMs (Figure 6A,C,E). IF and western blot analysis revealed significantly decreased pINSR activation in 3D OAdi a‐iCM compared to Adi a‐iCMs (p = 0.0122, Figure 6G). These findings indicate that while adipocyte hypertrophy does not significantly alter INSR expression, it impairs INSR phosphorylation in a‐iCMs, potentially leading to insulin insensitivity, a hallmark of diabetes mellitus [106].

Reduced insulin receptor autophosphorylation is an early defect seen in the skeletal muscle cells of fat‐fed, insulin‐resistant rats [107], and similarly, it was previously shown that elevated plasma FFA levels disrupted insulin signaling and glucose uptake in human skeletal muscle [108]. In the heart, animal studies showed that elevated circulating FFAs induced cardiac dysfunction and reduced myocardial glucose transporter expression [109], and that chronic fatty acid exposure impaired both basal and stimulated glucose uptake [110]. In vitro, PA treatment of human embryonic stem cell‐derived CMs for 16 h led to decreased insulin‐stimulated glucose and fatty acid uptake [111]. Here, we compared basal and insulin‐stimulated glucose uptake of 3D a‐iCMs cultured with 3D Adi/OAdi for 5 days and observed that insulin significantly enhanced glucose uptake in 3D Adi a‐iCMs (p = 0.0002), whereas 3D OAdi a‐iCMs showed no significant insulin responsiveness (Figure 6B). Basal and insulin‐stimulated glucose uptake was significantly higher in 3D Adi a‐iCMs compared to 3D OAdi a‐iCMs (p = 0.0252, p = 0.0308, Figure 6B), suggesting that obese adipocytes impair both basal and insulin‐stimulated glucose uptake in a‐iCMs. Here, we report that even without additional fatty acid supplementation to the CMs, palmitate‐treated adipocytes are sufficient to disrupt insulin signaling and impair glucose uptake in CMs. Under normal physiological conditions, lean adipocytes promote CM's insulin sensitivity by secreting anti‐inflammatory adipokines like adiponectin and omentin, which support glucose metabolism and reduce oxidative stress in the heart [8, 35]. Consistent with this, our results showed that incorporating lean adipocytes into a‐iCMs culture significantly enhanced insulin‐stimulated glucose uptake compared to a‐iCMs alone (*p*<0.0001, Figure 6B). Interestingly, a‐iCMs cultured alone did not exhibit a significant increase in glucose uptake upon insulin stimulation, which may be due to their high basal reliance on glycolysis (Figure 2J) and limited insulin responsiveness in the absence of adipocyte‐derived factors, an observation that warrants further investigation.

### 2.8 3D Hypertrophic Adipocytes Increase Endoplasmic Reticulum Stress and Promote Intracellular Fat Accumulation in a‐iCMs

The ER is a central organelle for protein folding, lipid synthesis, and calcium homeostasis in CMs [112]. Under physiological conditions, the ER ensures proper posttranslational folding and trafficking of membrane and secretory proteins [113], while maintaining calcium signaling critical for excitation‐contraction coupling [114]. However, when exposed to metabolic stressors such as excess FFA, the folding capacity of the ER becomes stressed [115], leading to an accumulation of misfolded or unfolded proteins. In an attempt to regulate ER stress, cells activate the unfolded protein response (UPR) primarily through ER stress sensors such as protein kinase R‐like ER kinase (PERK) [116]. While UPR initially promotes cellular adaptation by enhancing chaperone expression, reducing protein translation, and increasing ER‐associated degradation, chronic or unresolved ER stress drives maladaptive responses, including oxidative stress, inflammation, calcium mishandling, and apoptosis, contributing to cardiac dysfunction. We hypothesized that the OAdi cultured a‐iCMs, cultured in an environment with high FFAs and proinflammatory cytokines such as IL‐6 and MIF, would have higher ER stress. Western blot analysis showed a significant increase in the phosphorylated PERK (pPERK)/PERK ratio in OAdi a‐iCMs compared to both Adi a‐iCMs (p = 0.0488, Figure 6H), indicating that hypertrophic adipocyte co‐culture increases ER stress sensor activation in a‐iCMs.

PERK/ATF4‐dependent pathway has been identified in CMs exposed to hyperglycemic and hyperlipidemic conditions, where ER stress contributes to myocardial inflammation in obese mice [117]. To further assess downstream UPR activation in our system, we examined the PERK‐mediated integrated stress response, including ATF4 and CHOP expression. We observed a modest increase in ATF4 in OAdi a‐iCMs compared to Adi a‐iCMs (Figure S14). We next quantified CHOP, a key pro‐apoptotic transcription factor induced under prolonged ER stress. CHOP/β‐actin expression remained relatively low and comparable across all groups (Figure S14), indicating that the level and/or duration of ER stress over this acute 5‐day co‐culture period might not be sufficient to trigger overt engagement of the terminal pro‐apoptotic UPR arm.

Consistent with the 3D OAdi ConM treated a‐iCMs (Figure 3H,I), a‐iCMs cocultured with 3D OAdi had significantly increased ectopic lipid accumulation compared to control 3D a‐iCMs (p = 0.0471, Figure 6I,J). In a clinical study, it was found that patients with impaired glucose tolerance and T2D exhibit significant myocardial triglyceride accumulation, known as cardiac steatosis, a condition considered a preclinical marker of diabetic cardiomyopathy [118]. This lipid overload occurs early in the disease course, before the onset of diabetes‐related myocardial dysfunction, and is strongly associated with visceral adiposity rather than BMI or serum lipids [118]. In our study, by excluding exogenous lipids and exposing a‐iCMs only to lipids derived from the adipose depot, we demonstrated that hypertrophic‐adipocyte‐derived factors alone are sufficient to induce cardiac steatosis. This lipid buildup likely reflects enhanced FFA uptake and ectopic fat deposition within 3D a‐iCMs cultured with 3D OAdi, which may exacerbate lipotoxic stress and contribute to ER stress, metabolic dysfunction, and alter cardiac function.

### 2.9 Paracrine Signatures of Hypertrophic Adipocytes‐a‐iCM Model Reflect the Proinflammatory Phenotype of the Obese Heart

To assess the paracrine factors secreted by the engineered tissues, we analyzed the secretome profiles of 3D Adi a‐iCM and 3D OAdi a‐iCM constructs using a human cytokine array (Figure 6K). IL‐6, IL‐8, MIF, and MCP‐1/CCL2 were markedly increased in the 3D OAdi a‐iCM secretome, which are reported to be elevated in the circulation of obese individuals compared to lean subjects and are linked to low‐grade inflammation, insulin resistance, atherosclerosis, and CVD [119, 120, 121, 122]. Additionally, cathepsins were modestly upregulated in 3D OAdi a‐iCM constructs, consistent with reports of increased cathepsin expression under conditions of cardiac stress, remodeling, and dysfunction [123]. Circulating cathepsin D levels are significantly increased in newly diagnosed type 2 diabetes, correlating with insulin resistance and early cardiac dysfunction [124], while elevated cathepsin L levels are associated with increased cardiovascular mortality in older adults [125]. These findings suggest that the 3D OAdi a‐iCM model recapitulates some key paracrine features of the obese heart, highlighting its relevance for studying obesity‐associated cardiac dysfunction.

### 2.10 Metformin Mitigates Hypertrophic Adipocyte‐Induced Impairments in a‐iCMs

#### 2.10.1 Metformin Increases Beating Velocity and Decreases Beating Frequency Variation of 3D OAdi a‐iCMs

**FIGURE 7:**
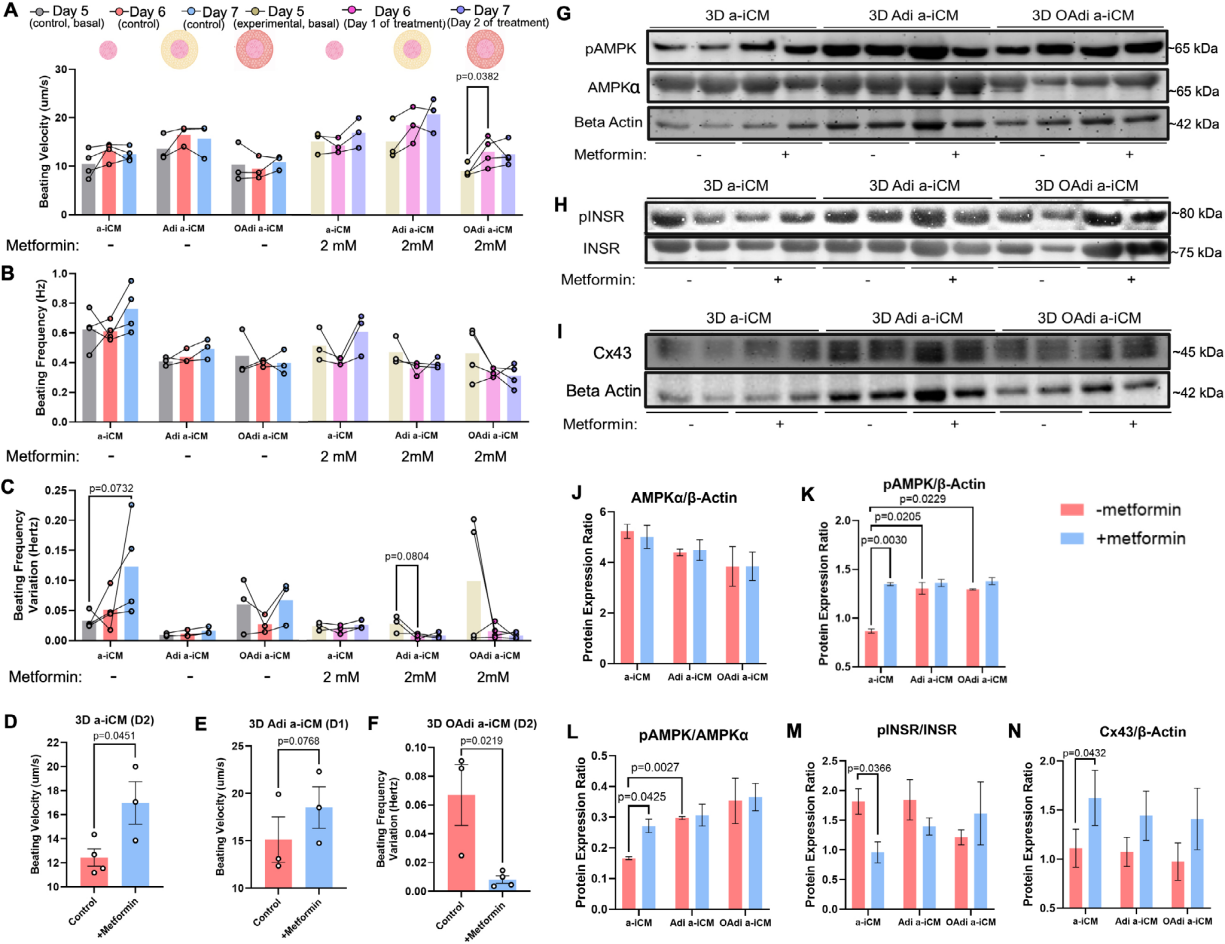
Metformin increases beating velocity and decreases beating frequency variation in OAdi‐a‐iCM constructs, indicating enhanced cardiac function. (A) Beating velocity (n = 3‐4, mean ± SEM; paired two‐tailed *t*‐test) (B) Beating frequency (n = 3‐4, mean ± SEM) and (C) Beating frequency variation of 3D constructs treated with 2 mM metformin (n = 3‐4, mean ± SEM; paired two‐tailed *t*‐test) (D) Beating velocity comparison of ‐/+ metformin 3D a‐iCM constructs (n = 3‐4, mean ± SEM; paired two‐tailed *t*‐test) (E) Beating velocity comparison of ‐/+ metformin 3D Adi a‐iCM constructs (n = 3, mean ± SEM; paired two‐tailed *t*‐test) (F) Beating frequency variation comparison of ‐/+ metformin 3D OAdi a‐iCM constructs (n = 3‐4, mean ± SEM; paired two‐tailed *t*‐test) (G) AMPKα, pAMPK and β‐actin western blots. (H) INSR and pINSR (I) Connexin43 (Cx43) and Beta Actin western blots. Western blot protein expression quantification of (J) AMPKα/β‐actin (n = 2, mean ± SD), (K) pAMPK/β‐actin (n = 2, mean ± SD; unpaired two‐tailed *t*‐test), (L) pAMPK/AMPK (n = 2, mean ± SD; unpaired two‐tailed *t*‐test), (M) pINSR/INSR (n = 4‐10, mean ± SEM), and (N) Cx43/β‐actin (n = 4‐10, mean ± SEM; unpaired two‐tailed *t*‐test).

Animal models of CVD have shown that metformin improves cardiac function, primarily through activation of AMPK [126, 127, 128]. To assess the therapeutic potential of metformin in mitigating OAdi‐induced dysfunction of a‐iCMs, we treated the constructs with 2 mM metformin for 48 h. Metformin significantly improved beating velocity in OAdi‐a‐iCM constructs after 24 h compared to basal levels (p = 0.0382, Figure 7A), suggesting enhanced contractile function. In 3D a‐iCM constructs, metformin significantly increased beating velocity after 2 days of treatment compared to controls (no treatment) (p = 0.0451, Figure 7D). In 3D Adi‐a‐iCM constructs, metformin led to a trend toward increased beating velocity 24 h after treatment (p = 0.0768, Figure 7E). Beating frequency across the construct groups (a‐iCM, Adi‐a‐iCM, OAdi‐a‐iCM) remained relatively unchanged between untreated and metformin‐treated conditions over 48 h (Figure 7B). While a‐iCM constructs in no‐treatment group after 48 h showed a modest increase in beating frequency variation, (p = 0.0732) and Adi‐a‐iCM constructs after 24 h of treatment showed a substantial decrease in beating frequency variation following metformin treatment (p = 0.0804, Figure 7C). OAdi‐a‐iCM constructs exhibited higher basal beating frequency variation compared to other groups, which was markedly reduced following metformin treatment (Figure 7C). In 3D OAdi‐a‐iCM, metformin significantly reduced beating frequency variation after 2 days compared to control (no treatment) (p = 0.0219, Figure 7F), a key metric associated with arrhythmias. This suggests that metformin may help mitigate beating irregularities, aligning with previous reports showing its association with a reduced risk of new‐onset atrial fibrillation in patients with obesity and T2D [129].

#### 2.10.2 Metformin Activates AMPK Signaling in 3D a‐iCMs

Metformin treatment led to notable activation of AMPK signaling across the constructs (Figure 7G). While total AMPKα protein levels normalized to β‐actin remained relatively unchanged with metformin in a‐iCM, Adi‐a‐iCM, and OAdi‐a‐iCM constructs (Figure 7J), pAMPK/β‐actin levels increased following treatment, particularly in a‐iCMs (p = 0.0030, Figure 7K). A similar trend was observed in Adi‐a‐iCMs and OAdi‐a‐iCMs with a modest increase in average pAMPK/β‐actin (Figure 7K). Importantly, the pAMPK/AMPKα ratio also significantly increased in 3D a‐iCM (p = 0.0425), indicating that metformin enhances AMPK activity rather than its expression (Figure 7L). While a modest increase in AMPK activation was observed in Adi‐a‐iCMs, these changes did not reach statistical significance (Figure 7L). Significant effects of metformin on beating velocity of a‐iCMs (p = 0.0451, Figure 7D) and the reduction in beating frequency variation in OAdi‐a‐iCMs (p = 0.0219, Figure 7F) may be mediated through AMPK pathway activation. Interestingly, basal pAMPK/β‐actin of 3D a‐iCMs was significantly lower than a‐iCMs in cocultures (p = 0.0205, p = 0.0229, Figure 7K). AMPK activation of a‐iCMs cultured with 3D Adi was significantly higher than a‐iCM cultured alone (p = 0.0027, Figure 7L). This finding could suggest an active metabolic crosstalk between adipocytes and a‐iCMs, likely driven by adipokine signaling (particularly adiponectin [130]). These results underscore the ability of adipocytes, both lean and hypertrophic, to modulate AMPK signaling and CM metabolism, and metformin to increase AMPK activity in 3D a‐iCMs.

#### 2.10.3 Metformin Alters Insulin Signaling and Gap Junction Expression of 3D a‐iCMs

In liver cells, metformin has been shown to increase insulin receptor activation, particularly with IRS‐2 signaling [131], while in skeletal muscle cells, it was reported to increase insulin‐stimulated tyrosine phosphorylation of the insulin receptor and IRS‐1 [132]. After metformin treatment, INSR activation levels in OAdi a‐iCM showed a marked increase (Figure 7M). Interestingly, INSR activation in 3D a‐iCMs was significantly lower after metformin treatment (p = 0.0366, Figure 7M). This could be due to AMPK activation reducing the reliance on INSR signaling, as previous studies have shown that AMPK activation can independently promote GLUT4 translocation to the cell membrane through insulin‐independent pathways [47]. However, this mechanism in CMs warrants further investigation.

It was previously reported that metformin can restore connexin40 and Cx43 concentrations in a murine atrial fibrillation model [133]. Given the improvement in cardiac function observed with metformin treatment, we analyzed the expression of Cx43, which was previously shown to be decreased in 3D OAdi a‐iCM constructs (Figure 5I,J). Metformin treatment significantly increased Cx43 expression in a‐iCM constructs compared to untreated controls (p = 0.0432) and led to a substantial increase in Adi/OAdi a‐iCM constructs, however, no significant changes in Cx43 expression were observed in these groups (Figure 7N).

Taken together, metformin treatment increased beating velocity and reduced beating frequency variation in 3D OAdi‐a‐iCM constructs, with a significant increase in beating velocity after 24 h and frequency variation after 48 h. In 3D OAdi a‐iCMs, these functional benefits were accompanied by a modest increase in AMPK and INSR activation as well as Cx43 expression. Collectively, these findings highlight metformin's ability to mitigate hypertrophic adipocyte‐induced impairments in CM function, possibly through AMPK activation, modulation of insulin signaling, and preservation of gap junction integrity. Additionally, significant increase in beating velocity, AMPK activation and Cx43 expression were observed in a‐iCM cultures alone, indicating that metformin's functional and metabolic benefits are evident even in the absence of adipocyte co‐culture.

## 3 Conclusions and Future Works

Despite the recognized importance of fatty acid component in cardiac tissue culture [76], there is a notable absence of human‐derived 3D models that incorporate both cardiac and adipose tissue elements. This gap in research limits our ability to fully understand the interactions between adipose tissue and myocardium in various (patho)physiological contexts, highlighting the need for the development of more comprehensive in vitro models. Here, we engineered a human‐derived, 3D bioprinted fat‐myocardium model designed to replicate the anatomical interface between EAT and atrial myocardium in obesity. For this, we first engineered 3D bioprinted hypertrophic adipocyte constructs that recapitulated key obesity hallmarks, including enlarged lipid droplets, altered cytoskeleton, increased lipolysis, proinflammatory cytokine release, impaired insulin signaling, and glucose uptake. We then investigated paracrine effects of hypertrophic adipocytes on a‐iCMs using the secretome of 3D OAdi, which induced metabolic inflexibility in a‐iCMs, marked by reduced mitochondrial respiration, glycolytic capacity, and ATP production, and was also accompanied by decreased beating velocity and reduced confluency compared to the lean group. Then, we integrated 3D a‐iCMs with the adipocyte constructs to create a concentric, spatially defined coculture system. This model allowed for controlled paracrine and physical interactions while preserving the ability to independently retrieve and analyze each tissue component without reliance on single‐cell sequencing or advanced sorting techniques. The 3D coculture revealed that direct interaction with hypertrophic adipocytes impaired a‐iCM beating kinetics with reduced beating frequency and confluency, and downregulated the gap junction protein Cx43 and insulin receptor activation, resembling key features of obesity‐induced atrial dysfunction. Metformin treatment increased beating velocity and reduced beating frequency variation in 3D OAdi‐a‐iCM constructs, as well as leading to a modest increase in AMPK, Cx43 and INSR activation. Overall, human‐derived 3D bioprinted fat‐myocardium model successfully recapitulates key structural, metabolic, and electrophysiological features of obesity‐induced atrial dysfunction and demonstrates metformin's ability to mitigate the changes in contractile and metabolic function of a‐iCMs cultured with hypertrophic adipocytes.

Future iterations of our model could benefit from the integration of iPSC‐derived adipocytes, which offer a renewable and scalable alternative to primary ADSCs. Unlike ADSCs, whose adipogenic potential declines with passaging, iPSCs enable consistent adipocyte generation and hold promise for modeling EAT‐myocardium interactions and offer high‐throughput drug screening. Additionally, enhancing the maturity of hiPSC‐derived CMs remains a critical avenue for improvement. Although these cells provide chamber specificity, they retain fetal‐like structural and metabolic characteristics that may limit their fidelity in modeling adult atrial pathophysiology [76]. Efforts to promote iCM maturation, such as mechanical/electrical stimulation [134, 135] or tailored metabolic maturation media [76], could improve their translational relevance. Additionally, future iterations of the model could be made more (patho)physiologically relevant by inducing hypertrophy with a mixture of fatty acids [136, 137], as palmitate alone does not fully represent the complex lipid composition of human obesity, where saturated and unsaturated fatty acids coexist and exert distinct yet interconnected metabolic and inflammatory effects. Lastly, a similar coculture framework could be applied to ventricular CMs to define chamber‐specific responses to adipocyte hypertrophy and to model obesity‐related ventricular myocardial pathologies.

Although we confirmed that 3D OAdi constructs recapitulate obesity‐associated morphological and adipokine hallmarks across both subcutaneous and epicardial adipocytes (Figure S2), the co‐culture studies in this work were conducted employing subcutaneous ADSCs. Utilizing subcutaneous ADSCs supported the development of a high‐throughput platform by enabling scalable adipocyte derivation from a readily obtainable source with minimal donor morbidity, and facilitated iterative system optimization prior to transitioning to more limited clinical samples. Incorporation of human EAT‐derived SVFs in future studies may further refine the physiological relevance of the platform and enable direct comparison between epicardial and non‐epicardial fat depots to interrogate reported depot‐specific characteristics [138] as well as their roles in obesity‐induced myocardial dysfunction. Furthermore, in contrast to our study, which primarily investigated the effects of adipocytes on CMs, future work could investigate how CM‐derived factors, cardiokines, influence adipocyte function, including lipid metabolism, adipogenesis, and inflammatory signaling [139].

This work provides mechanistic insights into adipocyte dysfunction‐induced arrhythmia, mitochondrial impairment, and disrupted insulin signaling, and establishes a versatile platform for investigating obesity‐induced CVD. Beyond mechanistic insights, the model holds translational potential for evaluating therapeutic agents, as demonstrated by metformin screening, and for guiding personalized treatment strategies for obesity‐related atrial dysfunction.

## 4 Experimental Section

### 4.1 Human ADSC Culture and 3D Bioprinting

Primary ADSCs were isolated from the axilla, mid back, flanks, and central abdomen of patients as described previously [140]. Isolation and characterization of ADSCs were carried out at Indiana University School of Medicine by Dr. Nakshatri's group, and the procedures to collect human subcutaneous adipose tissue were approved by the IU School of Medicine Institutional Review Board. Details on the characteristics of the ADSCs or SVFs used in this study, including donor BMI, gender, and other relevant information, are provided in Table S1.

GelMA hydrogels were synthesized utilizing an established protocol [141], Human Skin Collagen type 1 (10 mg/ml, Humabiologics) and photoinitiator (2‐hydroxy‐4‐(2‐hydroxyethoxy)‐2‐methylpropiophenone, Sigma) were purchased. Degree of methacrylation was quantified using NMR, and GelMA lysine methylene area and gelatin lysine methylene area were calculated using MestreNova. The degree of methacrylation of synthesized GelMA hydrogels was determined as 54% (Figure S11) using the equation reported previously [142]. Mechanical properties of the bioink were characterized utilizing the frequency sweep test and nanoindenter, as reported before [44]. GelMA‐Collagen type 1 bioink had Young's modulus of 2.0 ± 0.8 kPa [44], which is within the reported value for both myocardium [143] and adipose tissue [144].

ADSCs were maintained in ADSC Growth Medium and trypsinized at ∼80% confluency. Collagen type I (1 mg/mL, final concentration) was combined with 5 million/mL ADSCs and mixed with photoinitiator (0.025%, final concentration) and GelMA (10%, final concentration). ADSC concentration (5 million/mL) was selected to approximate the physiological adipocyte density in healthy EAT, which we estimated from the reported mean diameter of healthy epicardial adipocytes (70 µm) [145]. Bioink was loaded into cartridges and extruded through 22G nozzles onto sterilized, charged glass slides (Globe Scientific Inc.) using the CELLINK BioX6 bioprinter and a custom‐generated G‐code. Constructs were bioprinted in a hollow cylindrical shape (4 mm outer diameter, 2 mm inner diameter, 0.3 mm height) following the parameters outlined in Table S2, and subsequently, constructs were photocrosslinked for 30 s under UV light (6.9 mW/cm^2^) using a UV lamp (Lumen Dynamics). Three days after bioprinting, ADSC constructs were differentiated using adipogenic induction media (Lonza) as explained previously [146], and lipid droplet accumulation was validated by Nile Red (Invitrogen) staining. After the adipocyte differentiation protocol (Figure S6A), the intracellular accumulation of lipid droplets in adipocytes was validated using Nile Red staining (Figure S1A) and BF imaging (Figure S1B). PA‐induced hypertrophy was performed using DMEM/F12 1%FBS supplemented with 750 µM PA pre‐conjugated to BSA (Cayman) to obtain hypertrophic adipocytes as described before [16]. Vehicle media supplemented with 80 µM of fatty acid‐free BSA (Sigma Aldrich, USA) was added to the control group. 3D Adi/OAdi were incubated in DMEM/F12 1% FBS for 6 days with media changes done every 2 days, and ConM was collected for further analysis.

Human EAT was obtained from a donor whose heart were not suitable for transplantation through the Indiana Donor Network (IDN) or the National Disease Research Institute (NDRI) (Supplemental Table S1). Institutional Review Board (IRB) oversight was waived because no identifying donor information was provided. All human tissue procurement followed the principles of the Declaration of Helsinki. EAT located between the visceral pericardium and the left atrial myocardium was collected and transferred into Hank's Balanced Salt Solution (HBSS) containing antibiotics. Using sterile forceps and scissors, the remaining non‐adipose tissue was removed, and enzymatic isolation of SVF cells was carried out by adapting established protocols [140, 147]. Briefly, human EAT were digested in HBSS containing 0.25% (w/v) collagenase type I (Sigma‐Aldrich) and 0.25 U/mL Liberase TH (Roche) under agitation for 3 h at 37°C. Then, the suspension was filtered through 100 µm cell strainer (Corning) and centrifuged the remaining suspension at 500g for 10 min. The pellet was resuspended in DMEM/F12 containing 10% FBS and centrifuged at 300g for 8 min. The cell pellet was treated with 1X red cell lysis buffer (BD Biosciences) for 10 min centrifuged at 300g for 8 min. Finally, the pellet was resuspended in ADSC maintenance media (Lonza).

### 4.2 Characterization of 3D Hypertrophic Adipocytes

24 h after fatty acid treatment, cells were stained with Nile Red to visualize intracellular lipid droplets, and the droplet sizes of 3D Adi and OAdi were visualized using fluorescence microscopy. TRITC‐labelled F‐actin (Invitrogen) immunostaining was conducted to visualize the cytoskeletal changes during PA‐induced hypertrophy. Basal lipolysis was quantified by incubating the scaffolds in DMEM/F12 for 2 days at 37°C. The conditioned media were collected, and the Glycerol Glo Assay kit (Promega, WI, USA) was utilized to quantify basal lipolysis rate following the manufacturer's protocol using a plate reader (SPARK plate reader, TECAN).

To quantify glucose uptake, 3D Adi/OAdi were washed and starved for 3 h (no FBS/no glucose). Then, 10 µM human insulin was added to the constructs for 1 h. 1 mM 2DG in PBS was added to each well and incubated for 10 min at room temperature. Glucose uptake by 3D Adi and OAdi was assessed using the Glucose Uptake‐Glo Assay Kit (Promega, WI, USA), and luminescence was recorded with a plate reader according to the manufacturer's instructions. Luminescence values of 2DG uptake were normalized to cell viability, which was quantified using the CellTiter‐Glo Luminescent Cell Viability Assay (Promega, WI, USA).

The relative cytokine content of 3D Adi/OAdi constructs was determined using the human XL cytokine array (R&D Systems, Cat. No: ARY028) and human adipokine array (R&D Systems, Cat. No: ARY024) kits following manufacturer's instructions. Briefly, cytokine array membranes were blocked with an array buffer for 1 h at room temperature, washed, and incubated overnight at 4°C in an equal volume of the conditioned media. The membranes were washed and then incubated with the biotinylated antibody cocktail solution for 1 h, followed by incubation in streptavidin‐horseradish peroxidase (HRP) for 30 min and the chemiluminescent reagent for 1 min. The membranes were then exposed to X‐ray for 15 min using a biomolecular imager (ImageQuant LAS4000, GE Healthcare). Relative cytokine levels were determined by quantifying the dot intensity using ImageJ. Commercial ELISA kits were used to quantify human adiponectin (KHP0041; Invitrogen, USA) and human leptin (KAC2281; Invitrogen, USA) following the manufacturer's protocols. Insulin receptor prevalance was assessed by western blot quantifying INSR protein. For western blot analysis, 3D constructs were snap‐frozen and crushed using liquid nitrogen. Then, the resulting powder was incubated with RIPA buffer with 1% proteinase inhibitor cocktail at 4°C for 30 min. Western blots were conducted by adapting established protocols [148, 149]. The protein concentration of each construct was quantified via bicinchoninic acid (BCA) assay (Thermo Fischer), and equal amounts of protein were separated by 10 or 12% sodium dodecyl sulfate‐polyacrylamide gel electrophoresis (SDS‐PAGE) at 200 V for 45 min and transferred to the polyvinylidene difluoride (PVDF) blotting membrane at 100 V for 1 hr or 70 V for 3 hrs. The PVDF membranes were blocked in SuperBlock Blocking Buffer (#37536; Thermo Fisher Scientific) for 1 h at room temperature and incubated at 4°C overnight with primary antibodies. After three Tris‐buffered saline with Tween20 washes for 10 min each, the membranes were incubated with goat HRP‐conjugated anti‐rabbit or mouse secondary antibodies (ab205718, Abcam, 1:1000) for 1 hr at room temperature. Membranes were then incubated with a chemiluminescent substrate (Clarity ECL, Bio‐Rad) for 3 min and imaged on a ChemiDoc‐It2 system (UVP, Analytik Jena) using VisionWorks software and the pixel density of each protein band was quantified using ImageJ. Blots were stripped using Restore Western Blot Stripping Buffer (#21059; Thermo Fisher Scientific) and reprobed several times to visualize other proteins.

### 4.3 Differentiation of hiPSC‐Derived Atrial Cardiomyocytes

SeVA1016 (reprogrammed from human skin fibroblasts) [150] hiPSCs (RRID: CVCL_UK18) were differentiated into a‐iCM and v‐iCM (for comparison) by adapting previously reported two‐week small molecule‐based treatment protocols (Figure S12A) [44, 151, 152]. For a‐iCM differentiation, once hiPSCs reached 80%–95% confluency (Day 0), cells were treated with CM (−) (RPMI Medium 1640, Corning) supplemented with B27 without insulin (2%, Gibco), beta‐mercaptoethanol (β‐ME) (final concentration of 0.1 mM, Promega) containing 5 µM CHIR99021 (Stemcell Technologies) for 48 h. On Day 2, the medium was replaced with CM (‐) supplemented with 5 µM IWP‐4 (Stemcell Technologies). On Day 3, 1 µM retinoic acid (Sigma‐Aldrich) was added without media aspiration, followed by a fresh medium change with CM (‐) supplemented with 1 µM retinoic acid on Day 4. On Day 6, the medium was replaced with CM (−). Starting from Day 8, cells were maintained in fresh CM (+) (RPMI Medium 1640 supplemented with B27 with insulin (2%, Gibco), β‐ME (final concentration of 0.1 mM), with media changes every 3 days. Beating of a‐iCMs and v‐iCMs was observed by Day 15 of differentiation. On Day 30, differentiation efficacy and chamber specificity of the a‐iCMs were assessed by immunostaining for sarcomeric alpha‐actinin, troponin T (TnT), and MLC2A markers, applying concentrations as recommended by the manufacturer (Supporting Information: Table S3), and following the immunostaining protocol outlined in our previous work [44]. MLC2A expression was characterized utilizing flow cytometry, using a previously established protocol [153]. IF results showed the striated cytoskeletal structures (Figure S13A) and aligned Z discs (Figure S13B). We characterized the chamber specificity of iCM cultures, using immunostaining (Figure S12B), flow cytometry (Figure S12C), and beating analysis (Figure S12D‐E). IF analysis revealed high expression of atrial cardiac marker MLC2A in a‐iCM and ventricular marker MLC2V in v‐iCM cultures (Figure S12B). Video analysis of lateral displacement of spontaneous beating chamber‐specific iCMs was generated using an in‐house MATLAB code with the block‐matching algorithm, as we previously described [154]. After differentiation, we analyzed the differentiation efficacy of iCMs by immunofluorescence and flow cytometry [155], and the functionality of iCMs by analyzing beating and electrophysiological properties utilizing BF video analysis [44]. Consistent with the literature [151], a‐iCMs had significantly higher beating frequency than v‐iCMs (Figure S12E). All cell cultures were routinely tested for mycoplasma contamination using a PCR‐based detection kit (MycoAlert, Lonza) and were confirmed to be mycoplasma‐negative.

### 4.4 Characterizing the Metabolic Effects of Hypertrophic Adipocytes on a‐iCM

Seahorse XF96 extracellular flux analyzer was used to assess mitochondrial function as we described before [156]. Briefly, a‐iCMs were seeded at a density of 100,000 cells per Matrigel‐coated well in Seahorse XF96 Microplates five days before the assay. 48 h prior to the assay, fresh control media containing 2% B27 Supplement (50X), minus antioxidants (Cat. No. 10889038, Thermo Fisher Scientific) were applied to a‐iCMs, while 3D Adi/OAdi ConM were similarly supplemented with 2% B27 minus antioxidants containing insulin before being applied to monolayer a‐iCMs. One hour prior to the assay, the culture medium was replaced with Agilent Seahorse XF RPMI Basal Medium supplemented with 2 mM glutamine, 10 mM glucose (no glucose for Glycolysis Stress Test), and 1 mM sodium pyruvate. For the Mito Stress test, the Seahorse XF Cell Mito Stress Test Kit (Agilent) was used, with inhibitors prepared at the following concentrations: oligomycin (2.5 µM), FCCP (2 µM), and a combination of rotenone and antimycin A (Rot/AA, 2.5 µM). For the Glycolysis Stress test, Seahorse XF Glycolysis Stress Test Kit (Agilent) was used, with inhibitors prepared at the following concentrations: glucose (10 mM), oligomycin (1 µM), and 2‐DG (50 mM). For Glycolytic Rate test, Seahorse XF Glycolytic Rate Assay Kit (Agilent) was used, with inhibitors prepared at the following concentrations: Rot/AA (0.5 µM) and 2‐DG (50 mM). The number of a‐iCMs per well was quantified using Hoechst 33342 (Thermo Scientific, 8 µM) after a 30 min incubation at 37°C, followed by image analysis with ImageJ. OCR, ECAR, and PER were then normalized to the cell count. Baseline OCR was determined as the average of measurements taken from point 1 to 3, Basal Respiration, Maximal Respiration, Spare Respiratory Capacity, Coupling Efficiency, ATP Production‐Coupled Respiration, MitoATP, Basal Glycolysis, Compensatory Glycolysis, GlycoATP, and % Proton Efflux Rate from Glycolysis were analyzed on Seahorse Analytics. Glycolytic Capacity (ECAR after oligomycin injection—ECAR before glucose injection), % Glycolytic Reserve ((Glycolytic Capacity—Basal Glycolysis) / Glycolytic Capacity) × 100), and Non‐Glycolytic Acidification (ECAR before glucose injection) were derived from the raw data. These values were subsequently graphed and analyzed using GraphPad Prism. In supplementary experiments, additional treatment groups, Adiponectin (Human Recombinant Adiponectin, Abcam) (low (8 ng/mL)/high (12 ng/mL)), Leptin (Human Recombinant Leptin, VWR) (low (3000 pg/ml)/high (5000 pg/ml)), and PA (BSA‐conjugated PA, Cayman) (low (250 µM)/high (500 µM)), were included, and a‐iCM treatments were conducted using the same time frame. Adiponectin and leptin concentrations were selected based on trends observed in ELISA results. The 3D Adi group was characterized by high adiponectin and low leptin levels, whereas the 3D OAdi group exhibited low adiponectin and high leptin levels (Figure S1F‐G). JC‐1 (Abcam, ab113850) was conducted as reported before [157], and CellROX (Thermo Fisher Scientific, C10444) assay were conducted in accordance with the manufacturers’ instructions.

### 4.5 Characterization of Paracrine Effects of Hypertrophic Adipocyte on a‐iCM

To analyze the paracrine effects of hypertrophic adipocyte on a‐iCM's contractile function, ConM treatment is detailed in Section 4.4. was repeated. After 48 h, 3D Adi and OAdi ConM‐treated a‐iCMs were compared in terms of beating velocity, frequency, and confluency using a video analysis generated using an in‐house MATLAB code with the block‐matching algorithm, as previously described [154]. Then, electrophysiological changes induced by hypertrophic adipocytes were further analyzed by characterizing the calcium (Ca^2^^+^) transient of a‐iCMs. Cells were washed with PBS, and the medium was replaced with a Ca^2^^+^‐sensitive Fluo‐4 AM (Life Technologies) solution, following the manufacturer's instructions. Real‐time beating videos of 3D a‐iCMs were recorded using a fluorescence microscope equipped with a Hamamatsu C11440 digital camera, with a 30 ms exposure time for 20 s. Acquisition of calcium transient videos were performed under blinded conditions with respect to 3D Adi versus 3D OAdi cultured a‐iCM identity, and recordings in which calcium transient peaks could not be detected or quantified, due to low signal amplitude, elevated noise, or motion artifacts, were excluded across all groups. The rate of Ca^2^^+^ release (time to peak Ca^2^^+^ transient amplitude) and calcium transient duration at 50% (CaTD50) and 90% (CaTD90) of the amplitude were derived from intensity versus time plots. CaTD50 and CaTD90 were normalized to the spontaneous beat rate using the Fridericia correction to account for differences in beating frequency across samples. Additionally, intracellular fat accumulation in a‐iCMs treated with 3D Adi/OAdi ConM was characterized by Nile Red staining and quantified in ImageJ.

### 4.6 Engineering of Hypertrophic Adipocyte a‐iCM Co‐Culture Model

To visualize cell colocalization and spatial distribution across tissue layers, ADSC‐adipocytes were tagged with CellTracker orange CMTMR (1 µM, Life Technologies). At Days 30‐35 of the differentiation, a‐iCMs were tagged with CellTracker far red (1 µM, Life Technologies) and detached using trypsin‐EDTA supplemented with 0.25 U/mL Liberase TH (Roche). Collagen type I was combined with a‐iCM pellet and mixed with photoinitiator and GelMA. Using a concentric design with an adipocyte shell surrounding the a‐iCM core (∼1:3) (Figure 4B), we employed a 90 million/ml a‐iCM bioink, yielding a cell ratio of roughly 1:6 (ADSCs:CMs), which aligns with reported histological assessments of the native EAT‐myocardium interface [158]. a‐iCM bioink was loaded into cartridges and extruded through 20G nozzles using the droplet bioprinting setting on the CELLINK BioX6 Bioprinter. The media was removed from the wells containing 3D bioprinted ADSC‐derived lean and obese adipocytes prior to the second round of bioprinting. The bioink was bioprinted as droplets into the core of the 3D Adi/OAdi constructs and subsequently photocrosslinked for 30 s under UV light (6.9 W/cm^2^) using a UV lamp. In parallel, 3D a‐iCM‐only control groups were bioprinted onto sterilized charged glass slides (Globe Scientific Inc.) as controls using the same a‐iCM bioink. All constructs were incubated in tailored coculture media with 5:1 RPMI Medium 1640:DMEM/F12 supplemented with 2% B27 with insulin. 24 h after bioprinting, the tile images were taken with a fluorescence microscope (Zeiss, Hamamatsu ORCA flash 4.0) and stitched together using Zen Software.

### 4.7 Characterization of the Hypertrophic Adipocyte a‐iCM Co‐Culture Model

Twenty‐four hours after the a‐iCM bioprinting and at Day 5 of coculturethe viability of the 3D Adi/OAdi‐a‐iCM models was analyzed utilizing Live‐Dead assay (Life Technologies) following the manufacturer's instructions, and live/dead cells were counted using ImageJ, and %viability was calculated as reported before [44]. Constructs were stained using Nile Red, TnT, and MLC2A to colocalize adipocytes and a‐iCM in the constructs. After five days in culture, a‐iCMs in both cultures were compared in terms of beating velocity, frequency, and confluency using BF beating video analysis as well as Ca^2^^+^ transient, as detailed in section 4.5. Cx43, INSR, pINSR, PERK, pPERK, ATF4, and CHOP levels were compared between groups using immunostaining and western blot analysis. For Western blot analysis, the a‐iCM and adipocyte components of the constructs were carefully separated on Day 5, snap‐frozen immediately, and stored at ‐80°C. For immunostaining, constructs were washed and fixed with 4% PFA, and immunostaining protocol was applied as explained previously (44). Glucose uptake of a‐iCMs cultured with 3D Adi/OAdi was conducted as detailed in Section 4.2. Intracellular fat accumulation in 3D a‐iCMs was characterized by Nile Red staining and quantified in ImageJ.

### 4.8 Metformin Treatment of Hypertrophic Adipocyte a‐iCM Co‐Culture Model

Five days after a‐iCM bioprinting, 3D a‐iCM (control), and Adi/OAdi‐a‐iCM were treated with 2 mM metformin (Sigma‐Aldrich, MO, USA) for 48 h. The treatment media was replaced after 24 h to maintain drug activity and nutrient availability. Following treatment, constructs underwent the same characterization protocol described in Section 4.5. Briefly, beating kinetics, and calcium transients were characterized as detailed in Section 4.5. To examine molecular changes, protein expression levels of Cx43, INSR, pINSR, pAMPK, and AMPK were analyzed by western blotting. For western blots, constructs were disassembled to separate the a‐iCM and adipocyte components, snap‐frozen, lysed, and analyzed as described in Sections 4.2. and 4.7.

### 4.9 Statistical Analysis

Pre‐processing steps: data cleaning, normalization, and assessment of outliers, were performed prior to analysis. Data are represented as mean ± standard deviation (SD) or mean ± standard error of the mean (SEM), as specified in each figure legend. The sample size (n) for each experiment or comparison is reported in the corresponding figure legends. Statistical comparisons among three or more groups were performed using one‐way analysis of variance (ANOVA) followed by Tukey's post hoc test. Differences between paired samples were compared by paired Student's *t*‐test. For comparisons between two unpaired groups, a two‐sided unpaired Student's *t*‐test was used. Analysis of leptin and adiponectin concentration, as well as the leptin/adiponectin ratio, was performed using multiple linear regression. *p*‐value of less than 0.05 (*p*<0.05) was considered statistically significant. All analyses were performed using GraphPad Prism and R.

## Author Contributions

L.E.C. and P.Z. conceptualized the manuscript. L.E.C. performed methodology, investigation and visualization. P.Z. performed supervision. L.E.C. wrote the original draft. L.E.C. and P.Z. wrote, reviewed, and edited the manuscript.

## Conflicts of Interest

The authors declare no conflicts of interest.

## Supporting information




**Supporting file**: advs74183‐sup‐0001‐SuppMat.pdf.

**Supporting file**: advs74183‐sup‐0002‐Movie S1.mp4.

**Supporting file**: advs74183‐sup‐0003‐Movie S2.mp4.

**Supporting File**: advs74183‐sup‐0004‐Movie S3.mp4.

**Supporting File**: advs74183‐sup‐0005‐Movie S4.mp4.

## Data Availability

The data supporting the findings of this study are available from the corresponding author upon reasonable request.
