# Supplementary material for: 3D Bioprinted Fat‐Myocardium Model Unravels the Role of Adipocyte Hypertrophy in Atrial Dysfunction
Source: Adv Sci (Weinh). 2026 Feb 8;13(23):e16114. doi: 10.1002/advs.202516114 (PMC13104139; doi:10.1002/advs.202516114)
Supplement: Supplementary file 1 — Supporting file: advs74183‐sup‐0001‐SuppMat.pdf. [file ADVS-13-e16114-s003.pdf]

Supplementary Materials

3D Bioprinted Fat-Myocardium Model Unravels the Role of Adipocyte Hypertrophy in Atrial Dysfunction

Lara Ece Celebi[1,2], *and Pinar Zorlutuna*[1,2,3,4]*

[1] Department of Aerospace and Mechanical Engineering, University of Notre Dame, Notre Dame, IN 46556
[2] Bioengineering Graduate Program, University of Notre Dame, Notre Dame, IN 46556
[3] Department of Chemical and Biomolecular Engineering, University of Notre Dame, Notre Dame, IN 46556
[4] Harper Cancer Research Institute, University of Notre Dame, Notre Dame, IN 46556

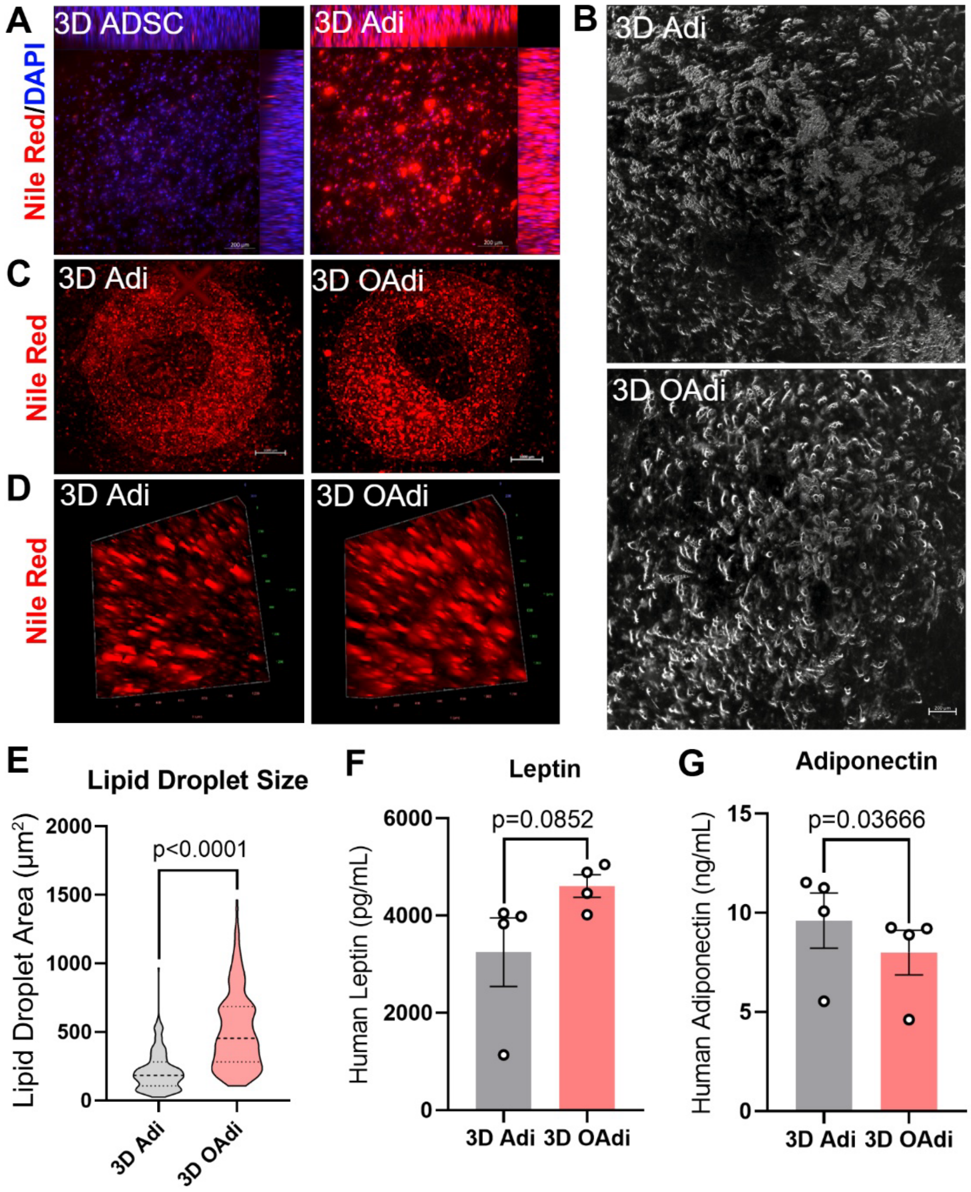

Supplementary Figure 1: Characterizations of 3D lean (3D Adi) and obese adipocyte (3D OAdi) contructs. A) Nile Red (lipid marker) staining of 3D ADSCs and 3D ADSC-derived adipocytes, B) Bright field images of 3D Adi and 3D OAdi *(scale bar=200 µm),* C) Nile Red IF images of 3D Adi and 3D OAdi *(scale bar=1000 µm),* and D) magnified images. E) Lipid droplet area quantification in 3D Adi/OAdi (≥176 lipid droplets quantified per group, mean ± SEM; unpaired two-tailed t-test). F) Leptin (n=4, mean ± SEM; batch-adjusted linear regression) and G) Adiponectin (n=4, mean ± SEM; batch-adjusted linear regression) concentrations of 3D Adi/OAdi secretome. Data represent mean ± SEM with individual data points shown.

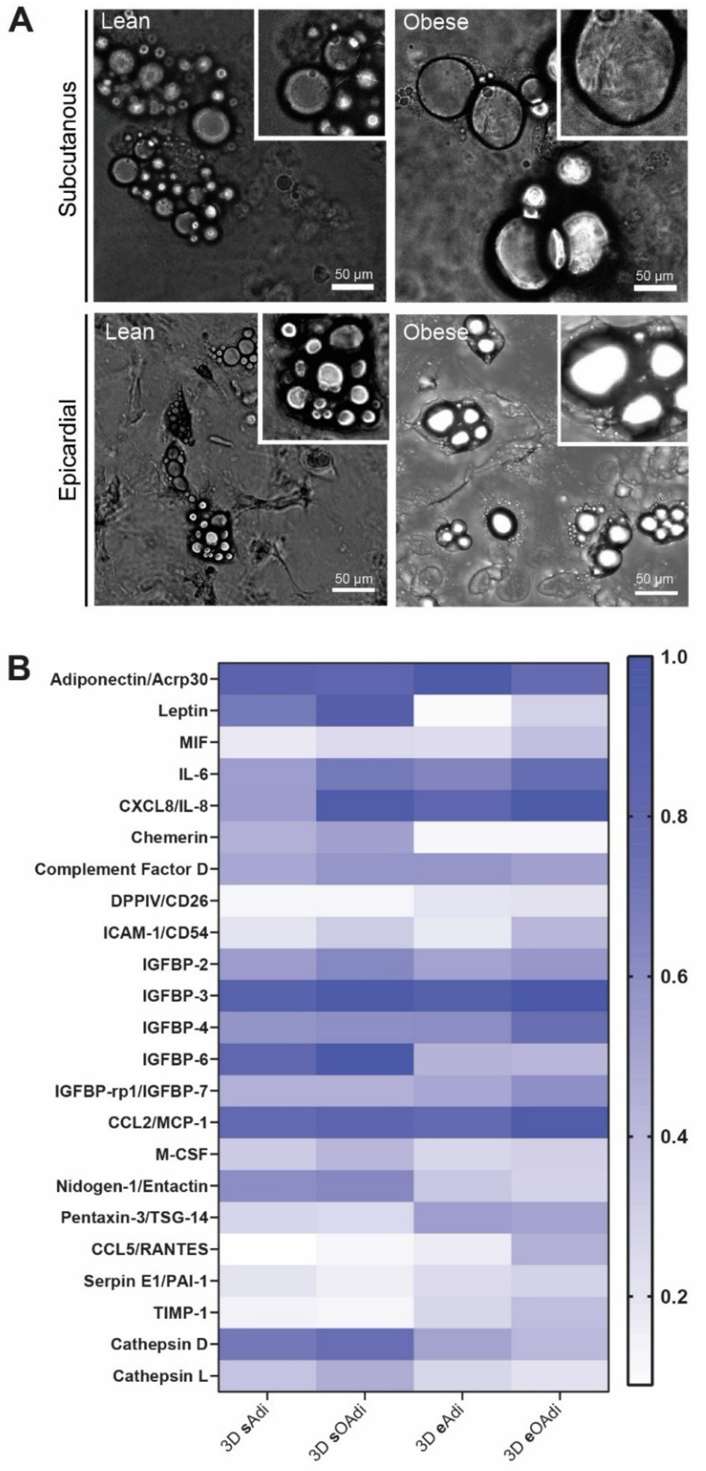

Supplementary Figure 2: Morphological and secretome comparison of subcutaneous (3D sAdi/sOAdi) and epicardial (3D eAdi/eOAdi) adipose tissue derived stem cell derived 3D adipocyte construct. A) Brightfield images of 3D subcutaneous and epicardial adipocytes under lean and hypertrophic conditions *(Scale bars: 50 µm)*. B) Heatmap representing adipokine array for 3D sAdi, 3D sOAdi, 3D eAdi, and 3D eOAdi.

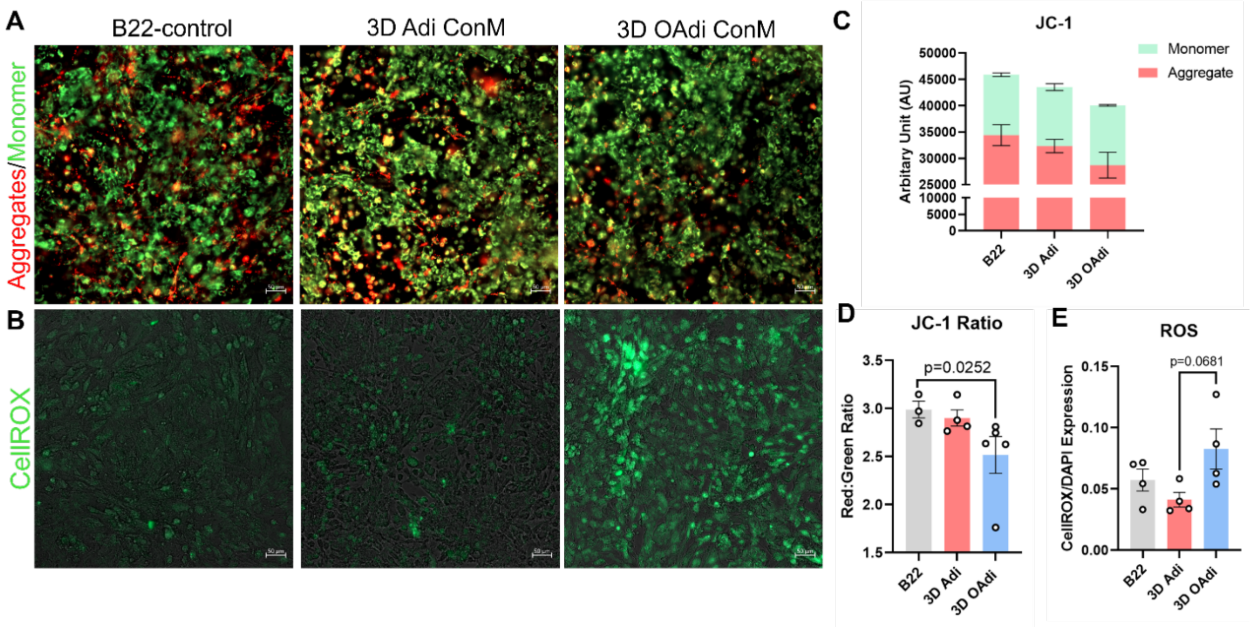

Supplementary Figure 3: Mitochondrial membrane potential and reactive oxygen assessment in a-iCMs treated with engineered construct conditioned media. A) Representative JC-1 staining images showing monomer (green) and aggregate (red) signals *(Scale bar=50 µm)*, and B) CellROX staining images for detection of reactive oxygen species in control (RPMI1640 supplemented with 2% B27 Supplement, minus antioxidants), 3D Adi conditioned media (ConM), and 3D OAdi ConM treated a-iCMs. C) Quantification of JC-1 monomer and aggregate fluorescence intensities *(Scale bar=50 µm)*, D) JC-1 red/green ratio across groups (n=3-5, mean ± SEM; One-way ANOVA with Tukey's post hoc test), and E) quantification of CellROX/DAPI expression across groups (n=4, mean ± SEM; One-way ANOVA with Tukey's post hoc test).

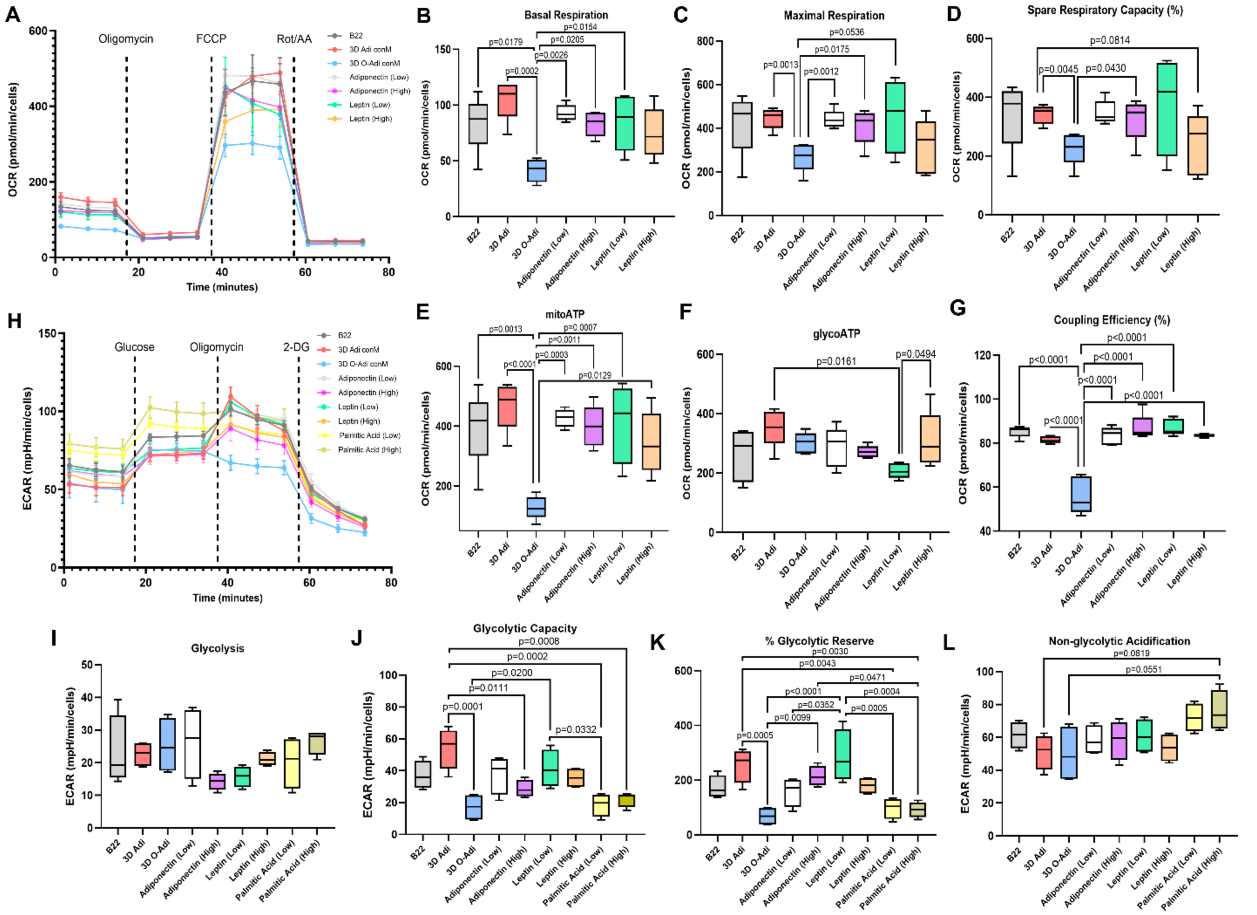

Supplementary Figure 4: Mitochondrial and glycolytic function analysis of a-iCMs treated with engineered construct conditioned media, low/high adiponectin, or low/high leptin. Extended Figure 2: A) Mito Stress test showing oxygen consumption rate (OCR) over time in response to: oligomycin (ATP synthase inhibitor), FCCP (uncoupler), and rotenone/antimycin A (Rot/AA, complex I/III inhibitors). B) Basal respiration (n=5) C) Maximal respiration (n=5) D) Spare respiratory capacity (%) (n=5) E) Mitochondrial ATP production (mitoATP) (n=5) F) Glycolytic ATP production (glycoATP) (n=4) G) Coupling efficiency of treated groups. (n=5) H) Real-time ECAR profile following sequential injections of glucose, oligomycin, and 2-deoxyglucose (2-DG) I) Glycolysis (n=4) J) Glycolytic capacity (n=4) K) Glycolytic reserve (n=4) L) Non-glycolytic acidification (n=4) Data are presented as mean ± SEM.

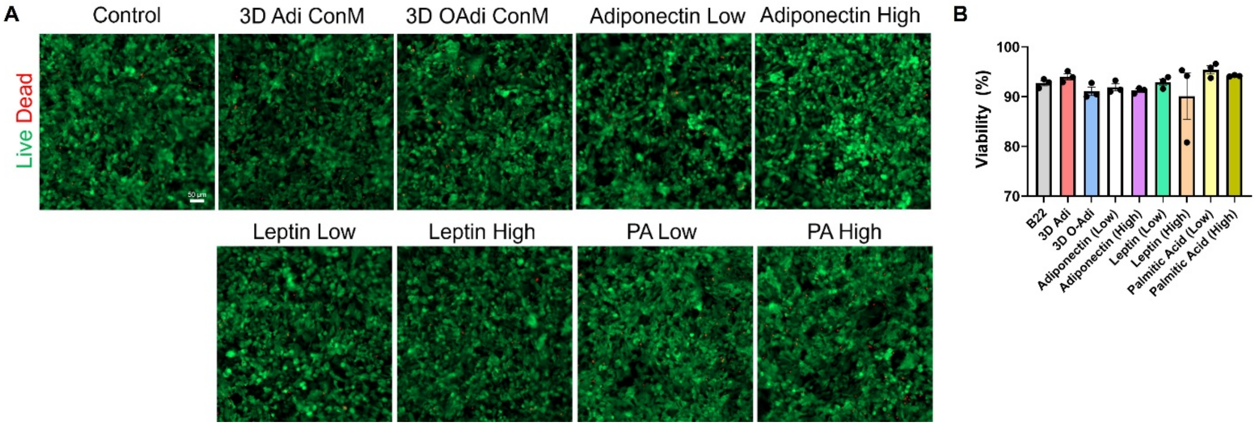

Supplementary Figure 5: Viability analysis of a-iCMs treated with engineered construct conditioned media, low/high adiponectin, low/high leptin and low/high palmitic acid (PA). A) Live-Dead (Green: live cells, red: dead cells) imaging (*Scale bar: 50 μm*) and B) viability quantification of a-iCMs after 48 hours of treatment (n=3). Data are presented as mean ± SEM.

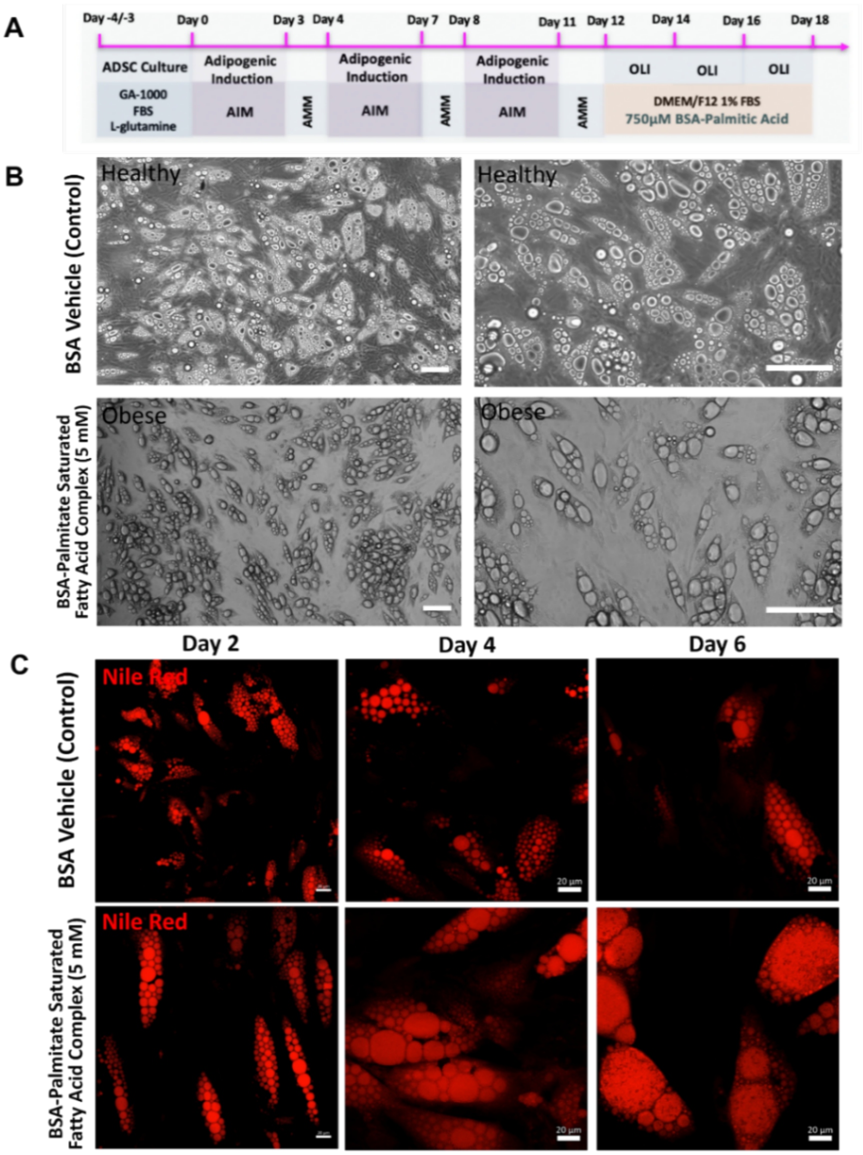

Supplementary Figure 6: ADSC-derived healthy and hypertrophic adipocyte characterization. A) Differentiation protocol of 2D/3D ADSC-derived adipocyte culture and fatty acid treatment (AIM: Adipogenic induction media, AMM: Adipogenic maintenance media, OLI: Obese like induction) B) Brightfield images of 2D healthy (Adi) and hypertrophic adipocyte (OAdi) cultures *(scale bar=100 µm)* C) Nile Red staining of Adi/OAdi on Days 2, 4 and 6 *(scale bar = 20 µm)*

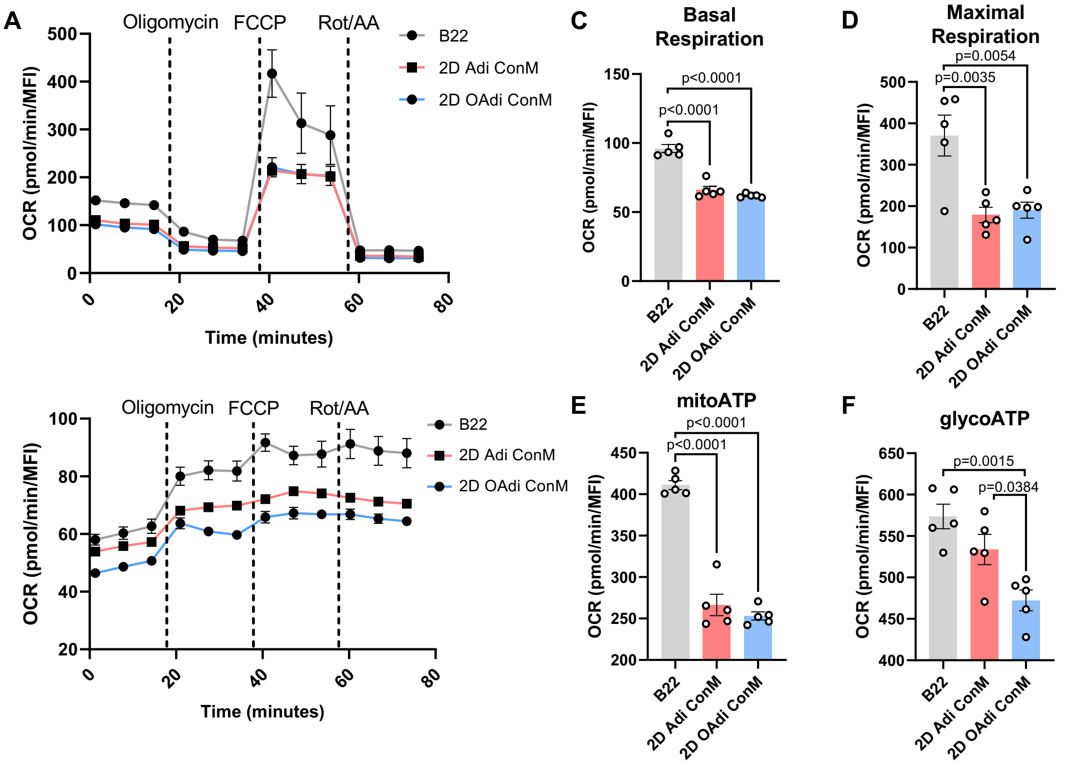

Supplementary Figure 7: Mitochondrial function analysis in a-iCMs treated with 2D Adi/OAdi conditioned media. A) Mito Stress test showing oxygen consumption rate (OCR) over time in response to: oligomycin, FCCP, and rotenone/antimycin A (n=5). B) Basal respiration (n=5) C) Maximal respiration (n=5) D) Mitochondrial ATP production (mitoATP) (n=5) E) Glycolytic ATP production (glycoATP) (n=5). Data are presented as mean ± SEM and one-way ANOVA with Tukey's post hoc test were used.

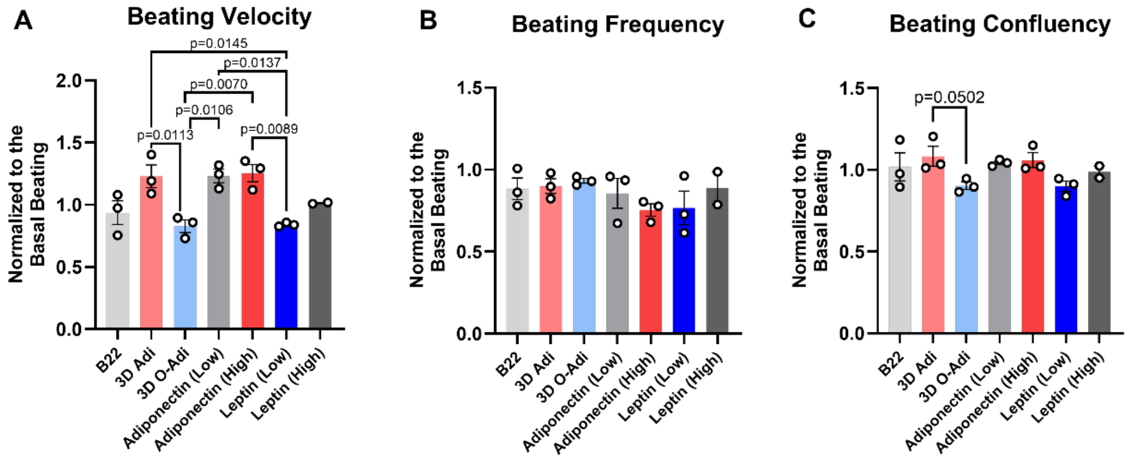

Supplementary Figure 8: Beating analysis of a-iCMs treated with engineered construct conditioned media, low/high adiponectin, or low/high leptin. Extended Figure 3A-C: A) Beating velocity (n=2-3) B) Beating frequency (n=2-3) C) Beating Confluency of the treated a-iCMs (n=2-3). Data are presented as mean ± SEM.

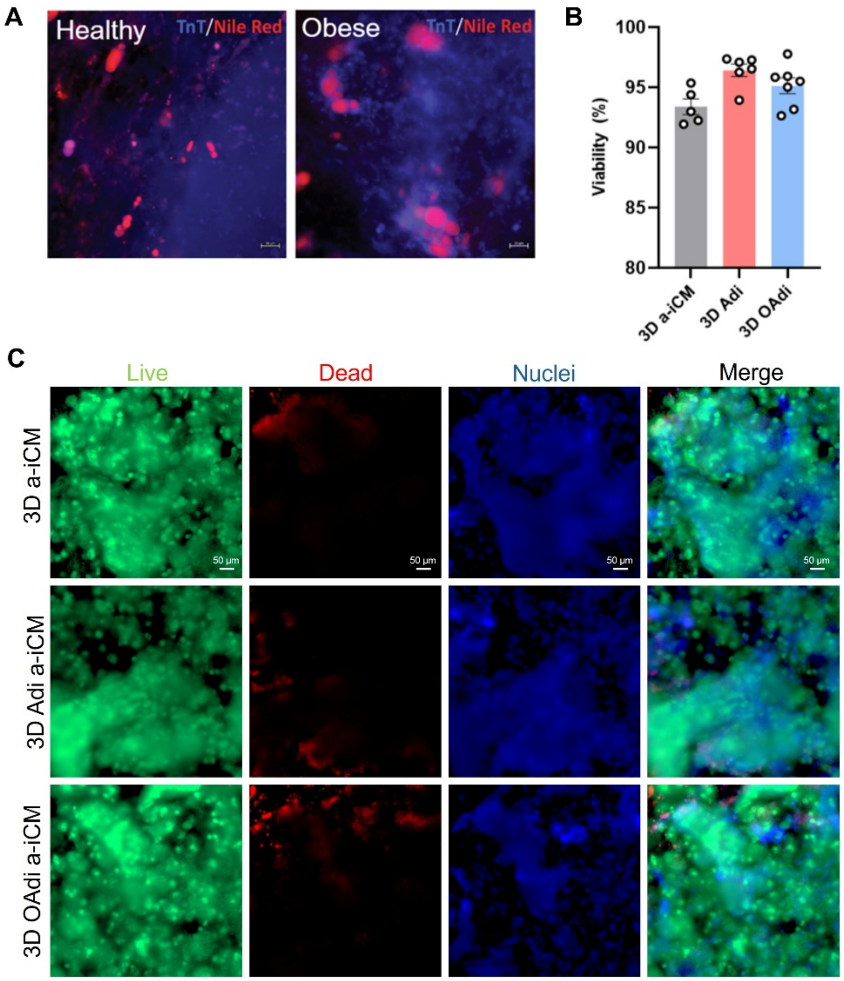

Supplementary Figure 9: Cell colocalization and viability in 3D bioprinted constructs. A) Immunofluorescence images of iCM marker (Troponin T (TnT), blue) and adipocyte marker (Nile Red, red) ***(Scale bar: 50 µm)*** at Day 1 of co-culture, B) Viability of constructs 24h after a-iCM bioprinting (n=5-7, mean ± SEM.), C) **Extended Figure 4H:** Viability of a-iCM cultured alone, with 3D Adi or 3D OAdi five days after 3D bioprinting **(Green: live cells, red: dead cells, blue: nuclei (hoechst 33342),** ***Scale bar: 50 µm*****)** .

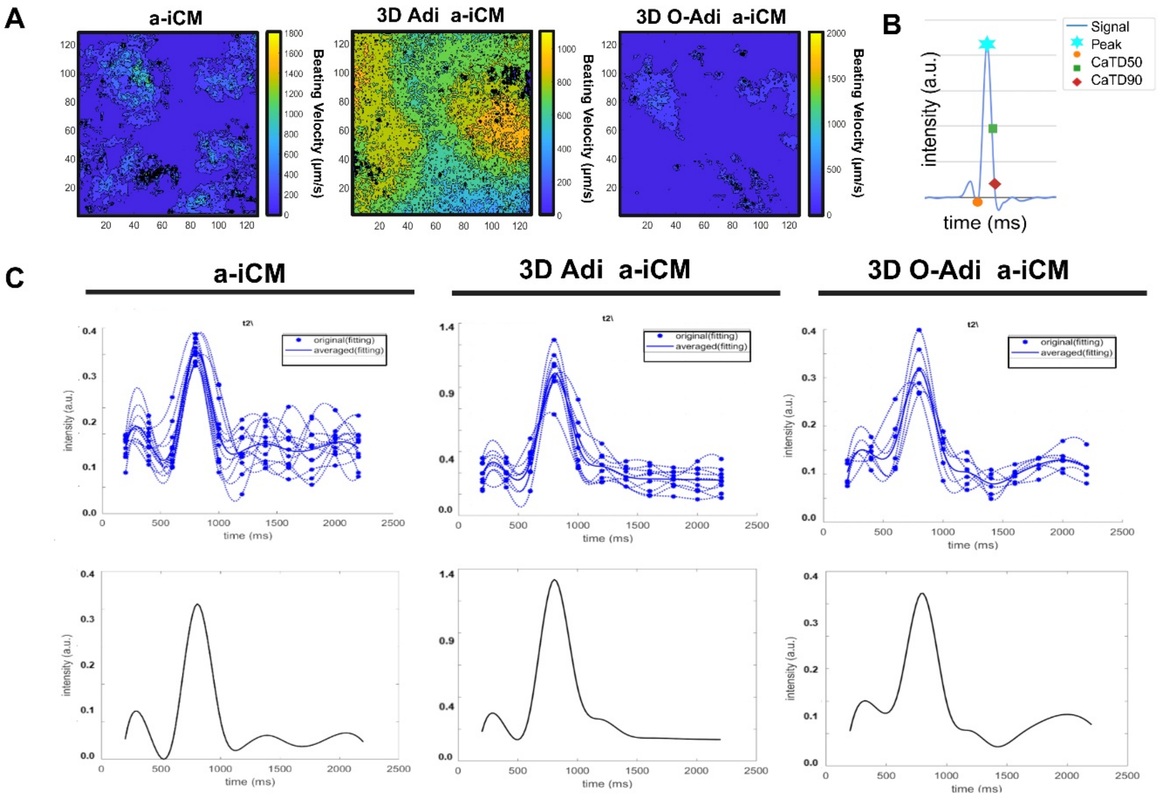

Supplementary Figure 10: Beating velocity mapping and Calcium Flux waveform analysis of 3D constructs. A) Heatmaps of beating velocity in 3D a-iCM constructs cultured alone (a-iCM) or with 3D Adi or 3D OAdi. B) Schematic of intensity (arbitrary units (a.u.))-time (milliseconds (ms)) plot showing a single calcium transient. (cyan=peak, orange=start, green=50% decay, red=90% decay). D) Calcium waveform overlays of 3D a-iCM constructs cultured alone (a-iCM) or with 3D Adi or 3D OAdi.

Supplementary Table 1: Characteristics of subcutanous human ADSCs and human epicardial adipose tissue-derived stromal vascular fraction cells used in the study.

Patient Number	Age	BMI	Gender	Site of Collection
1	34	26	Female	Axilla, mid back, flanks, central abdomen
2	31	20	Female	Flanks
3	36	22	Female	Abdomen, hips
4	7	18.85	Female	EAT located on left atrial myocardium

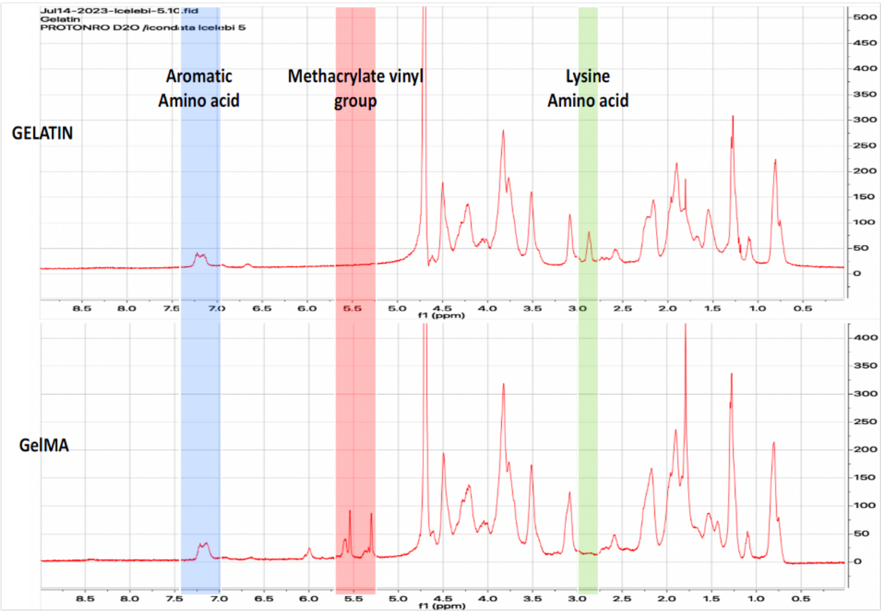

Supplementary Figure 11: H-NMR of gelatin and GelMA highlighting the aromatic amino acid (blue), methacrylate vinyl group (red), and lysine amino acid (green) peaks.

Supplementary Table 2. Bioprinting parameters of 3D constructs

Region	Printer	Nozzle Tip	Speed	Pressure	Temperature	Diameters
Adipocyte	CELLINK BioX6	22G (410 µm)	3-5 mm/s	12-30 psi	24-30°C	Concentric circle, 8 mm outer, 4 mm inner diameter, 0.3 mm height
a-iCM		20G (580 µm)				Droplet, 4 mm diameter, 0.3 mm height

Supplementary Table 3. Concentration and supplier information regarding the primary/secondary antibodies and chemical stains

Antibody/Stain	Concentration	Supplier	Catalog Number
Sarcomeric alpha-actinin (SAA)	1:200	Abcam	ab9465
Anti-cTnT antibody [1C11] mouse mAb	1:200	Abcam	ab8295
Vimentin	1:200	Abcam	ab8978

MLC2A	IF, FC:1:200	Synaptic Systems	311 011
MLC2V	IF, FC:1:200	Proteintech	10906-1-AP
Nile Red	2 µM	Molecular Probes	N1142
INSR	IF 1:200<br>WB 1:1000	Abcam	ab137747
Phospho-INSR (Tyr972)	IF 1:200<br>WB 1:1000	Abcam	44-800 G
Connexin 43 / GJA1 pAb	IF 1:200<br>WB 1:1000	Abcam	ab11370
Connexin 43	WB 1:1000	Cell Signaling	3512S
PERK	WB 1:1000	Cell Signaling	3192S
Phospho-PERK (Thr980)	WB 1:1000	Cell Signaling	3179S
Rhodamine Phalloidin	IF 1:400	Invitrogen	R415
Cell Tracker® Orange	1 µM	Thermo Fisher	C34551
CellTracker® Deep Red	1 µM	Thermo Fisher	C34565
Hoechst 33342	8 µM	Thermo Fisher	62249
AMPK Alpha	WB 1:1000	Thermo Fisher	PA5-105297
pAMPK (Thr172)	WB 1:1000	Thermo Fisher	PA5-37821
ATF4	WB 1:1000	Santa Cruz Biotechnology	sc-390063
CHOP	WB 1:1000	Santa Cruz Biotechnology	sc-71136

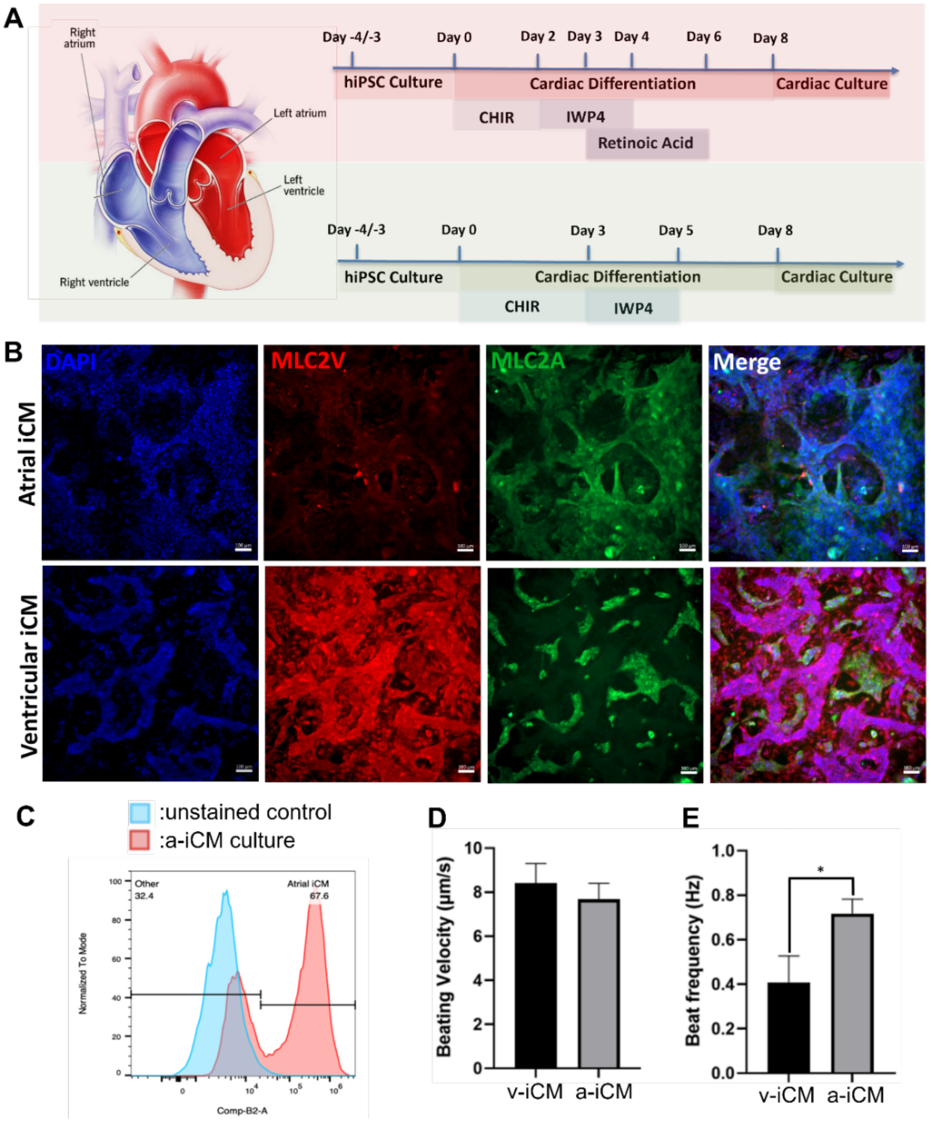

Supplementary Figure 12: Differentiation and characterization of hiPSC-atrial cardiomyocytes (a-iCM) A) Differentiation protocol of a-iCMs and hiPSC-ventricular cardiomyocytes (v-iCMs) B) MLC2A (atrial marker)/MLC2V (ventricular marker)/DAPI immunostaining of a-iCM and v-iCM cultures C) Flow showing MLC2A-positive a-iCM population D) Quantification of beating velocity (n=3) and E) frequency in chamber-specific iCMs, showing significantly higher beat frequency in a-iCMs (*$p < 0.05$) compared to v-iCMs (n=3) .

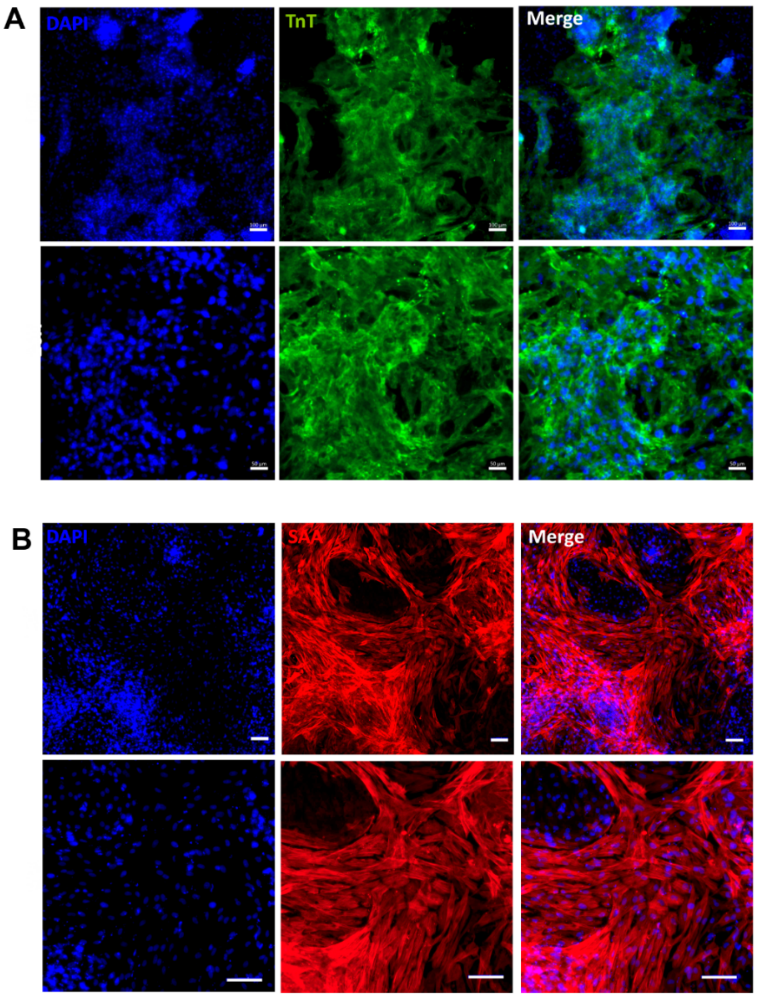

Supplementary Figure 13: Cardiac and cardiac muscle marker characterization of a-iCM culture A) Cardiac Troponin T (TnT, cardiac cell marker) *(scale bar=100 µm)* and close up images *(scale bar=50 µm)* B) Sarcomeric alpha-actinin (SAA, muscle cell marker) characterization of a-iCM culture *(scale bar=100 µm)* and close up images *(scale bar=100 µm)*

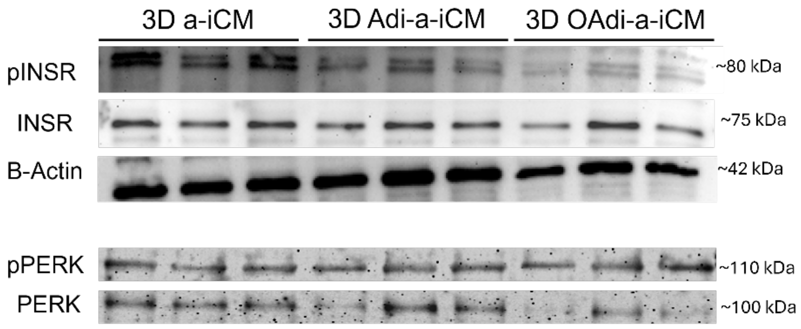

Supplementary Figure 14. Extended western blot images in Figure 6C–D showing pINSR INSR, B-actin, pPERK and PERK expressions, including 3D a-iCM-only control.

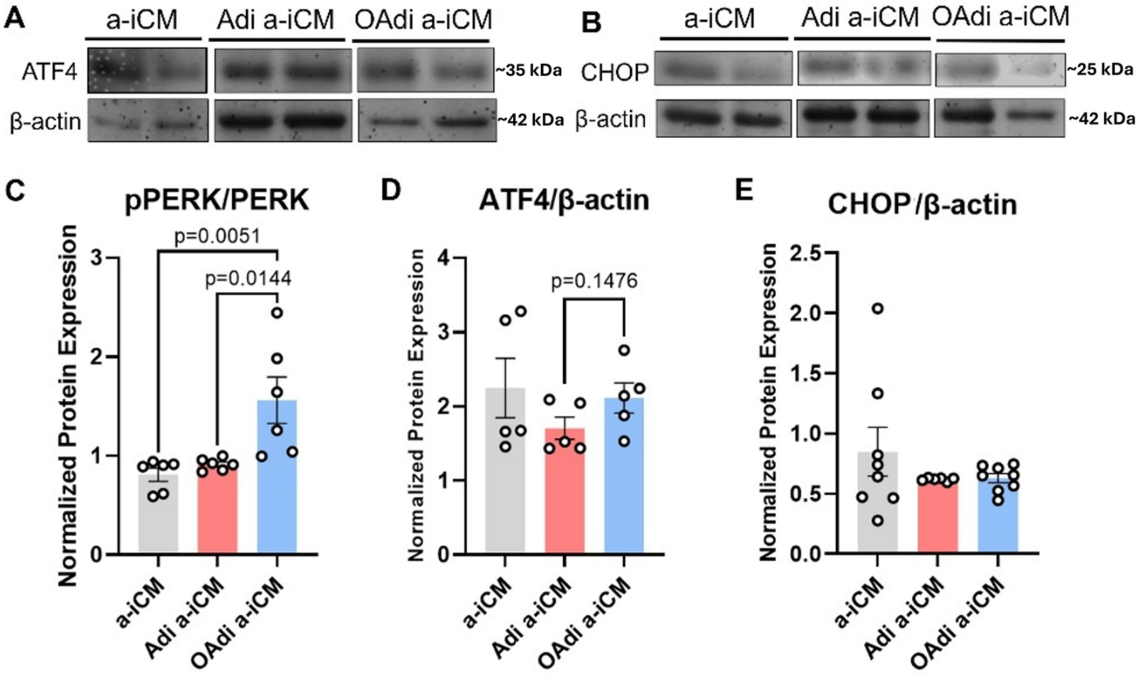

Supplementary Figure 15: Characterization of endoplasmic reticulum (ER) stress marker expression in 3D constructs. A) Western blots showing ATF4 and β-actin B) CHOP and β-actin expressions in a-iCM-only, 3D Adi-a-iCM, and 3D OAdi-a-iCM constructs. C) **Extended figure of Figure 6E:** PERK activation (n=6, mean ± SEM; One-way ANOVA with Tukey's post hoc test), D) ATF4 normalized to β-actin (n=5, mean ± SEM; One-way ANOVA with Tukey's post hoc test). E) CHOP normalized to β-actin (n=6-8, mean ± SEM) in 3D a-iCM, 3D Adi-a-iCM, and 3D OAdi-a-iCM constructs.

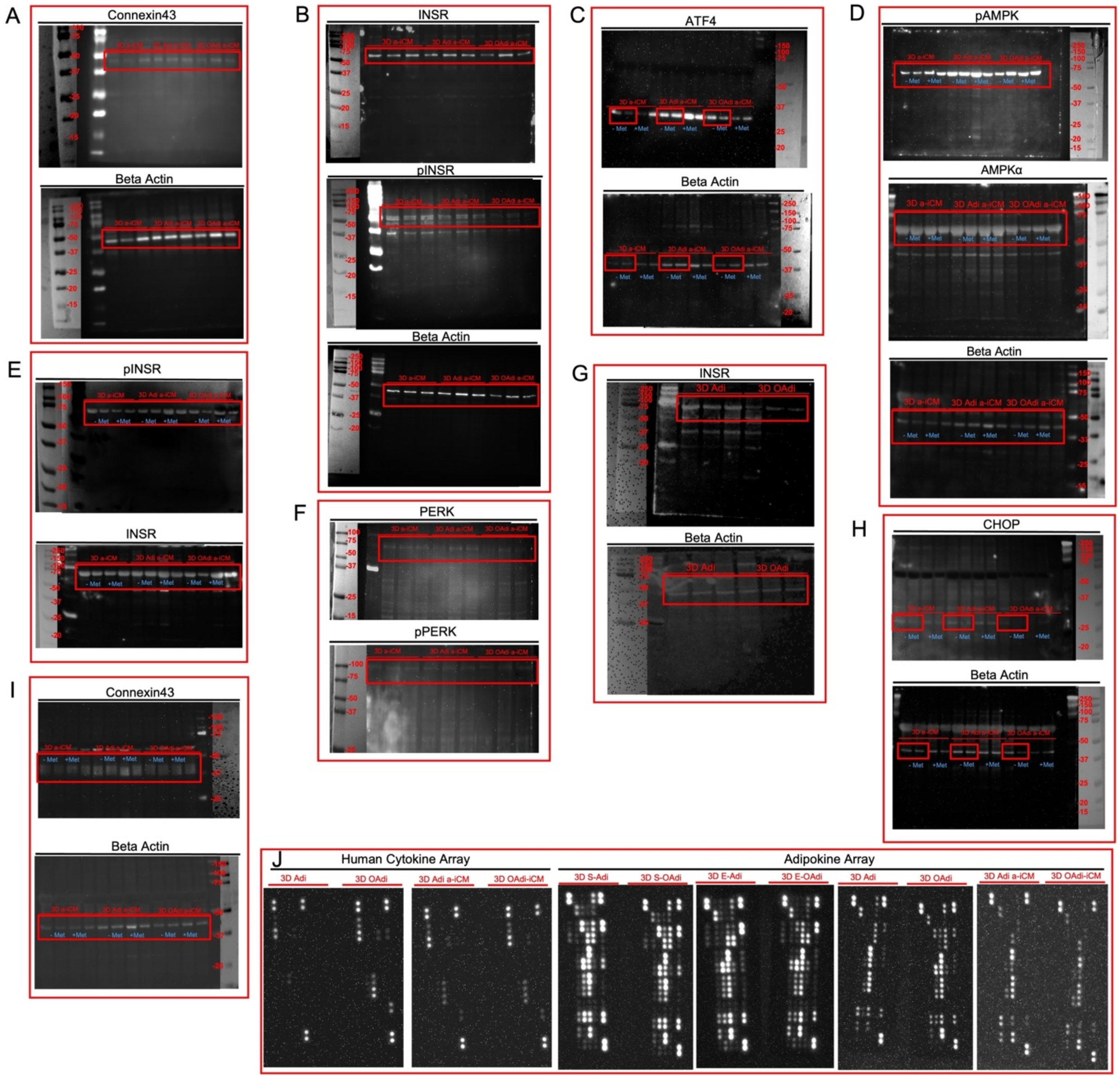

Supplementary Figure 16: Uncropped Western blots A) Connexin43 and Beta-actin of 3D a-iCM, Adi/OAdi a-iCMs; B) insulin receptor (INSR), phospho-INSR (pINSR) and Beta-actin of 3D a-iCM, Adi/OAdi a-iCMs; C) ATF4 and Beta-actin of 3D a-iCM, Adi/OAdi a-iCMs; D) AMPKα and phospho-AMPK (pAMPK) and Beta Actin of 3D a-iCM, Adi/OAdi a-iCMs; E) pINSR and INSR of 3D a-iCM, Adi/OAdi a-iCMs; F) PERK and phosphor-PERK (pPERK) of 3D a-iCM, Adi/OAdi a-iCMs; G) INSR and Beta Actin of 3D Adi/OAdi; and H) CHOP and Beta Actin of 3D a-iCM, Adi/OAdi a-iCMs; I) Connexin43 and Beta-actin of 3D a-iCM, Adi/OAdi a-iCMs; Red rectangles indicate the regions used for figure presentation and densitometric quantification, molecular weight markers (kDa) and lane identities (3D a-iCM, 3D Adi/OAdi a-iCM, 3D Adi/OAdi; ±Met) are annotated on each membrane. J) Membranes for the human cytokine array and adipokine array of 3D subcutaneous and epicardial ADSC Adi/OAdi and 3D Adi/OAdi a-iCM.

Supplementary Movie Captions

Supplementary Movie 1: Representative brightfield beating videos of 3D a-iCM, 3D Adi a-iCM, and 3D OAdi a-iCM constructs at Day 5 of co-culture. Video was recorded in real time under bright-field mode using an Axio Observer.Z1 microscope (Zeiss) equipped with a Hamamatsu C11440 digital camera for 20s intervals (Scale bar=50 µm).

Supplementary Movie 2: Representative calcium transient recordings of a-iCM treated with control media or 3D Adi/OAdi conditioned media for 48h (Scale bar=50 µm). Video was recorded in real time using a fluorescent Axio Observer.Z1 microscope (Zeiss) equipped with a Hamamatsu C11440 digital camera for 20s intervals (Scale bar=50 µm).

Supplementary Movie 3: Brightfield beating video of 3D OAdi a-iCM constructs at Day 5 of co-culture showing beating irregularity (skipped beat indicated by an arrow). Video was recorded in real time under bright-field mode using an Axio Observer.Z1 microscope (Zeiss) equipped with a Hamamatsu C11440 digital camera for 40s intervals, with 15s shown in the video (Scale bar=50 µm).

Supplementary Movie 4: Calcium transient recording of 3D OAdi a-iCM at Day 5 of co-culture showing beating irregularity. Video was recorded in real time using a fluorescent Axio Observer.Z1 microscope (Zeiss) equipped with a Hamamatsu C11440 digital camera for 30s intervals.
